# Generation and in vitro functional characterization of anti-CD22 chimeric antigen receptor T cells derived from newly generated monoclonal antibody clones

**DOI:** 10.1371/journal.pone.0362221

**Published:** 2026-11-12

**Authors:** Wipawadee Ruengdej, Vichaya Chuthaphakdikun, Sirirut Jewmoung, Nithidol Sakunrangsit, Chatikorn Boonkrai, Trairak Pisitkun, Supannikar Tawinwung, Koramit Suppipat

**Affiliations:** 1 Faculty of Pharmaceutical Science, MSc Program in Research for Enterprise, Chulalongkorn University, Bangkok, Thailand; 2 Center of Excellence in Cellular Immunotherapy, Chulalongkorn University, Bangkok, Thailand; 3 Cell and Gene Therapy Manufacturing Center, King Chulalongkorn Memorial Hospital, Bangkok, Thailand; 4 Faculty of Medicine, Center of Excellence in Systems Biology, Chulalongkorn University, Bangkok, Thailand; 5 Thailand Hub of Talents in Cancer Immunotherapy, Bangkok, Thailand; 6 Department of Pharmacology and Physiology, Faculty of Pharmaceutical Sciences, Chulalongkorn University, Bangkok, Thailand; 7 Department of Research Affairs, Faculty of Medicine, Chulalongkorn University, Bangkok, Thailand; Indian Institute of Chemical Technology, INDIA

## Abstract

Chimeric antigen receptor (CAR) T-cell therapy has transformed the treatment of B-cell malignancies, but relapse after CD19-directed therapy remains a major challenge, often because of loss or downregulation of CD19 expression. CD22 is an attractive alternative target because it is expressed on malignant B cells, including in many cases of CD19-negative disease. In this study, we generated and characterized novel anti-CD22 monoclonal antibody–derived CAR constructs for in vitro evaluation. Four anti-CD22 monoclonal antibody clones were initially screened, and two clones, 24A7G6 and 29H9C8, were selected for CAR development based on their favorable binding and CAR surface expression profiles. Second-generation CAR constructs containing either a 4−1BB or CD28 costimulatory domain were generated and introduced into primary human T cells. The resulting CAR T cells were evaluated for expansion, immunophenotype, activation and inhibitory marker expression, cytokine secretion, short-term cytotoxicity, long-term co-culture activity, and sequential tumor rechallenge against CD22-positive Raji and Nalm-6 cells and CD22-negative K562 cells. Both 24A7G6- and 29H9C8-derived CAR T cells expanded efficiently, showed predominant CD8 + populations with naïve and central memory phenotypes, and mediated potent antigen-specific cytotoxicity against CD22-positive target cells, while showing no activity against CD22-negative cells. Following antigen stimulation, CAR T cells upregulated activation markers and showed modest increases in inhibitory receptors. In long-term co-culture and sequential rechallenge assays, all constructs retained antitumor activity under prolonged antigen exposure. Notably, the 24A7G6-CD28 CAR demonstrated enhanced sustained tumor control compared with the corresponding 4−1BB construct in extended assays, whereas short-term cytotoxicity was broadly comparable across constructs. These findings show that the newly generated 24A7G6 and 29H9C8 scFvs can function as effective targeting domains for CD22-directed CAR T-cell development and provide an in vitro basis for prioritizing lead constructs for further preclinical evaluation.

## Introduction

Chimeric antigen receptor (CAR) T-cell therapy has emerged as a transformative treatment for hematologic malignancies, particularly B-cell cancers such as acute lymphoblastic leukemia (ALL) and non-Hodgkin lymphoma (NHL) [1,2]. CD19-directed CAR T-cell therapies have achieved remarkable clinical efficacy, with complete remission rates exceeding 80–90% in patients with relapsed or refractory (R/R) B-ALL [3–6]. Despite these successes, a significant proportion of patients experience disease relapse, often due to the emergence of CD19-negative leukemic clones. Such antigen escape results from alternative splicing, point mutations, or lineage switching that downregulate or eliminate CD19 expression, enabling tumor cells to evade CAR T-cell recognition [5,7–12]. This major clinical limitation underscores the need to identify and validate alternative therapeutic targets capable of sustaining durable remission and preventing immune escape.

CD22 has emerged as a promising target antigen for CAR T-cell therapy owing to its stable and restricted expression on normal and malignant B cells. Unlike CD19, CD22 expression generally persists after CD19-directed therapy, making it an attractive target for patients with relapsed or refractory disease [9,13–15]. CD22 is a transmembrane sialoglycoprotein belonging to the Siglec family that modulates B-cell receptor (BCR) signaling and maintains immune tolerance [16,17]. Its consistent surface expression and internalization properties make it suitable for antibody-based or CAR-mediated targeting. Indeed, early-phase clinical trials using CD22-directed CAR T cells have demonstrated encouraging responses in patients who relapsed after CD19 CAR T-cell therapy. However, therapeutic challenges remain, including variable antigen density, limited in vivo persistence, and the potential for early exhaustion of effector T cells [9,18–20].

This study focuses on the generation and characterization of novel anti-CD22 monoclonal antibody (mAb) clones and their integration into second-generation CAR constructs. By comparing CARs derived from distinct scFv frameworks and costimulatory domains (4−1BB versus CD28), this work aims to optimize CD22-targeted CAR T-cell functionality and persistence. Advancements in CAR design and antibody engineering are expected to expand the therapeutic landscape for B-cell malignancies, offering improved efficacy and safety profiles for patients with limited treatment options.

## Materials and methods

### Screening of anti-CD22 monoclonal antibodies

Monoclonal antibodies against CD22 were generated in collaboration with the Center of Excellence in Systems Biology, Chulalongkorn University (CUSB) using standard hybridoma technology. Screening was performed to identify antibody clones with optimal binding specificity and affinity. Flow cytometry was used to assess the mean fluorescence intensity (MFI) and percentage of CD22-positive binding across various cell lines. Antibody affinity was further evaluated by determining equilibrium dissociation constants (K_D). Clones with the highest binding stability and specificity were selected for subsequent CAR design.

### Structural modeling and domain binding prediction

Single-chain variable fragment (scFv) sequences from four newly generated anti-CD22 antibodies (24A7G6, 29H9C8, 36B1E12, and 37B7G12) were modeled using AlphaFold v2.3 [21]. The full-length extracellular domain of human CD22, comprising seven immunoglobulin-like domains (d1–d7), was also modeled using the same platform. Each scFv model was individually aligned with the CD22 structure to predict domain-specific binding. Interactions were assessed based on proximity between complementarity-determining regions (CDRs) and CD22 residues (<5 Å). The predicted binding domains were annotated and visualized using PyMOL (Schrödinger, LLC).

### Cell lines and culture conditions

The following cell lines were used: Raji (RRID:CVCL_2699), Nalm-6 (RRID:CVCL_0092), K562 (RRID:CVCL_0004), Daudi (RRID:CVCL_0008), Molt-4 (RRID:CVCL_0013), THP-1 (RRID:CVCL_0006), and 293T (ATCC:CRL_3216). Cells were maintained in either Dulbecco’s Modified Eagle Medium (DMEM, high glucose; Cytiva) or RPMI-1640 medium (Cytiva) supplemented with 10% fetal bovine serum (FBS; Gibco), 1% GlutaMAX-I (Gibco), and 1% penicillin–streptomycin (HyClone, Cytiva). Cultures were incubated at 37 °C in a humidified atmosphere containing 5% CO_2_. Mycoplasma contamination was routinely monitored using the MycoAlert™ Mycoplasma Detection Kit (Lonza), and only mycoplasma-free cells were used.

### CAR construct Design and retroviral production

To generate the CAR construct, the scFv sequence derived from the selected anti-CD22 monoclonal antibody was cloned into a second-generation CAR backbone. The construct included a Strep-Tag II for transduction tracking, an IgG2 (CH3) hinge, a CD28 transmembrane domain, and intracellular signaling domains of either 4–1BB/CD3ζ or CD28/CD3ζ. For retrovirus production, 293T cells were co-transfected with 3.7 μg of the CAR plasmid, 2.5 μg of PegPam, and 3.7 μg of RDF packaging plasmids using GeneJuice® (Novagen). Supernatants were collected at 48 h and 72 h post-transfection, filtered through 0.45 μm syringe filters, and stored at −80 °C until use.

### Viral titration

Retroviral titers were determined by measuring CAR expression in transduced 293T cells using flow cytometry. Briefly, 50,000 293T cells were seeded per well in a 24-well plate. Serial viral dilutions (10 ⁻ ¹–10 ⁻ ⁴) were prepared in complete DMEM containing 8 μg/mL polybrene, followed by spinoculation at 2,000 × g for 10 min. After 72 h incubation, cells were analyzed for CAR expression. Transducing units per milliliter (TU/mL) were calculated as:

### Flow cytometry and antibodies

The following fluorochrome-conjugated antibodies were used: PE anti-CD22 (HIB22), APC anti-CD22 scFv (ACRO), FITC anti-Strep Tag II (5A9F9), PerCP anti-CD3 (OKT3), APC anti-CD3 (OKT3), APC anti-CD4 (OKT4), PE anti-CD8 (SK1), APC anti-CD45RO (UCHL1), FITC anti-CCR7 (DREG-56), APC anti-CD69 (FN50), PE anti-TIGIT (A151536), APC anti-TIM-3 (F38-2E2), and FITC anti-PD-1 (EH12.2H7). Flow cytometry was performed using a BD Accuri™ C6 Plus cytometer (BD Biosciences), and data were analyzed with FlowJo v10 (TreeStar). Detailed flow cytometry gating strategies for CAR expression, phenotypic analysis, and cytotoxicity assays are provided in the Supplementary Figure 1 (S1 fig.).

### Transduction of primary human T cells

The protocol and written informed consent for obtaining peripheral blood from healthy donors were approved by the Institutional Review Board of the Faculty of Medicine, Chulalongkorn University (COA No. 0647/2024, IRB No. 334/64). Peripheral blood were collected from June 1^st^ 2024 to April 30^th^ 2025. Peripheral blood mononuclear cells (PBMCs) were isolated by density gradient centrifugation. T cells were activated for 72 h in culture medium containing IL-2 (100 IU/mL). Retroviral transduction was performed in RetroNectin®-coated 24-well plates using a multiplicity of infection (MOI) of 0.5. Spinoculation was carried out at 2,000 × g for 90 min. After centrifugation, the viral supernatant was removed, and 2 × 10^5^ activated T cells per well were added to medium supplemented with IL-7 (10 ng/mL) and IL-15 (5 ng/mL). CAR expression was analyzed four days post-transduction by flow cytometry before functional assays.

### Characterization of anti-CD22 CAR T cells

CAR surface expression was confirmed using anti-CD22 scFv and Strep-Tag II antibodies. T-cell proliferation was quantified on days 4, 7, and 11 post-transduction using hemocytometer counts. T-cell subsets were analyzed by flow cytometry for CD4⁺ and CD8 ⁺ populations and memory phenotypes (naïve, central memory, and effector memory) based on CD45RO and CCR7 expression. Activation (CD69, CD25) and exhaustion markers (PD-1, TIM-3, LAG-3, TIGIT) were also evaluated.

### Cytotoxicity assay

Cytotoxic activity was assessed using GFP ⁺ CD22 ⁺ target cell lines (Raji and Nalm-6) and CD22 ⁻ K562 cells. CAR T cells were co-cultured with targets at effector-to-target (E:T) ratios of 1:1, 1:2, and 1:4 for 24, 48, and 72 h. After incubation, cells were stained with 7-AAD to identify dead cells. Viable target cells (GFP ⁺ 7-AAD⁻) and T cells (CD3⁺) were quantified by flow cytometry. Cytotoxicity was calculated by comparing the percentage of live target cells in CAR-treated versus untreated wells.

### Cytokine release assay

To assess functional cytokine secretion, supernatants were collected after 24 h of co-culture (E:T = 1:1). Levels of IFN-γ, TNF-α, IL-2, IL-4, IL-6, and IL-10 were measured using the BD™ Cytometric Bead Array (CBA) Human Inflammatory Cytokine Kit according to the manufacturer’s protocol.

### Long-term co-culture assay

To investigate long-term functional persistence, 0.1 x 10^6^ effector cells (normalized CAR-T or NT) were co-cultured with 1 x 10^6^ Raji GFP^+^ cells (1:10 E:T ratio). The assay was performed in 6-well tissue culture plates with 5% FBS TexMACS medium, without exogenous cytokines. The co-cultured cells were subjected to cytotoxicity assay using flow cytometry at day 1, 4, 7, and 12. The cytotoxicity was analyzed by gating for 7-AAD^-^ CD3^+^ viable effector cells and 7-AAD^-^ GFP^+^ viable tumor cells. The absolute count of residual effector and target cells was determined by multiplying the total viable cell population by the percentage of 7-AAD^-^ CD3^+^ or 7-AAD^-^ GFP^+^ cells identified via flow cytometry.

### Tumor cell rechallenge assay

Four million effector cells (normalized CAR-T cells or NT) were co-cultured with Raji GFP^+^ cells at a 4:1 effector to target (E:T) ratio in 24-well G-rex plates (Wilson Wolf, Saint Paul, Minnesota, United States) with 5% FBS TexMACS medium, without exogenous cytokines. The co-culture cells were harvested every 4 days for cytotoxicity assay using flow cytometry and subsequently rechallenged with 1 × 10^6^ Raji cells. The percentages of 7-AAD^-^ CD3^+^ viable effector cells and 7-AAD^-^ GFP^+^ viable tumor cells were assessed to calculate the absolute count of residual effector and target cells at each round.

### Statistical analysis

All statistical analyses were performed using GraphPad Prism v8 (GraphPad Software, Inc.). One-way ANOVA was used to compare experimental groups, with p < 0.05 considered statistically significant. All experiments were conducted in at least three biological replicates, and data are presented as mean ± SD.

## Results

### Characterization of newly generated anti-CD22 monoclonal antibodies

Four anti-CD22 monoclonal antibody (mAb) clones—24A7G6, 29H9C8, 36B1E12, and 37B7G12—were generated using hybridoma technology. Surface plasmon resonance (SPR) analysis using recombinant CD22 protein revealed that all clones specifically bound CD22 but exhibited distinct kinetic profiles (Fig 1A). Clone 24A7G6 displayed a rapid association and dissociation rate, corresponding to nanomolar affinity. Clones 29H9C8 and 37B7G12 demonstrated slower dissociation rates, suggesting more stable interactions, whereas 36B1E12 showed intermediate binding kinetics. The kinetic constants (K_a, K_d, K_D) for each clone are summarized in Table 1. Specificity was confirmed by flow cytometry across a panel of hematopoietic cell lines (Fig 1B). All clones bound strongly to CD22 ⁺ B-cell lines (Daudi and Raji) and weakly to Molt-4 cells, consistent with lower CD22 expression. No binding was detected in the CD22 ⁻ monocytic line THP-1, confirming antigen specificity.

**Table 1:** Kinetic binding parameters of anti-CD22 monoclonal antibody clones.

Clone	K_a_(1/Ms)	K_d_(1/s)	K_D_(M)
24A7G6	2.706 x 10^5^	2.940 x10^-4^	1.086 x 10^−9^
29H9C8	2.419 x 10^5^	1.541 x 10^−4^	6.370 x 10^−10^
36B1E12	9.590 x 10^4^	5.335 x 10^−5^	5.563 x 10^−10^
37B7G12	9.391 x 10^4^	4.704 x 10^−5^	5.006 x 10^−10^

**Fig 1:**
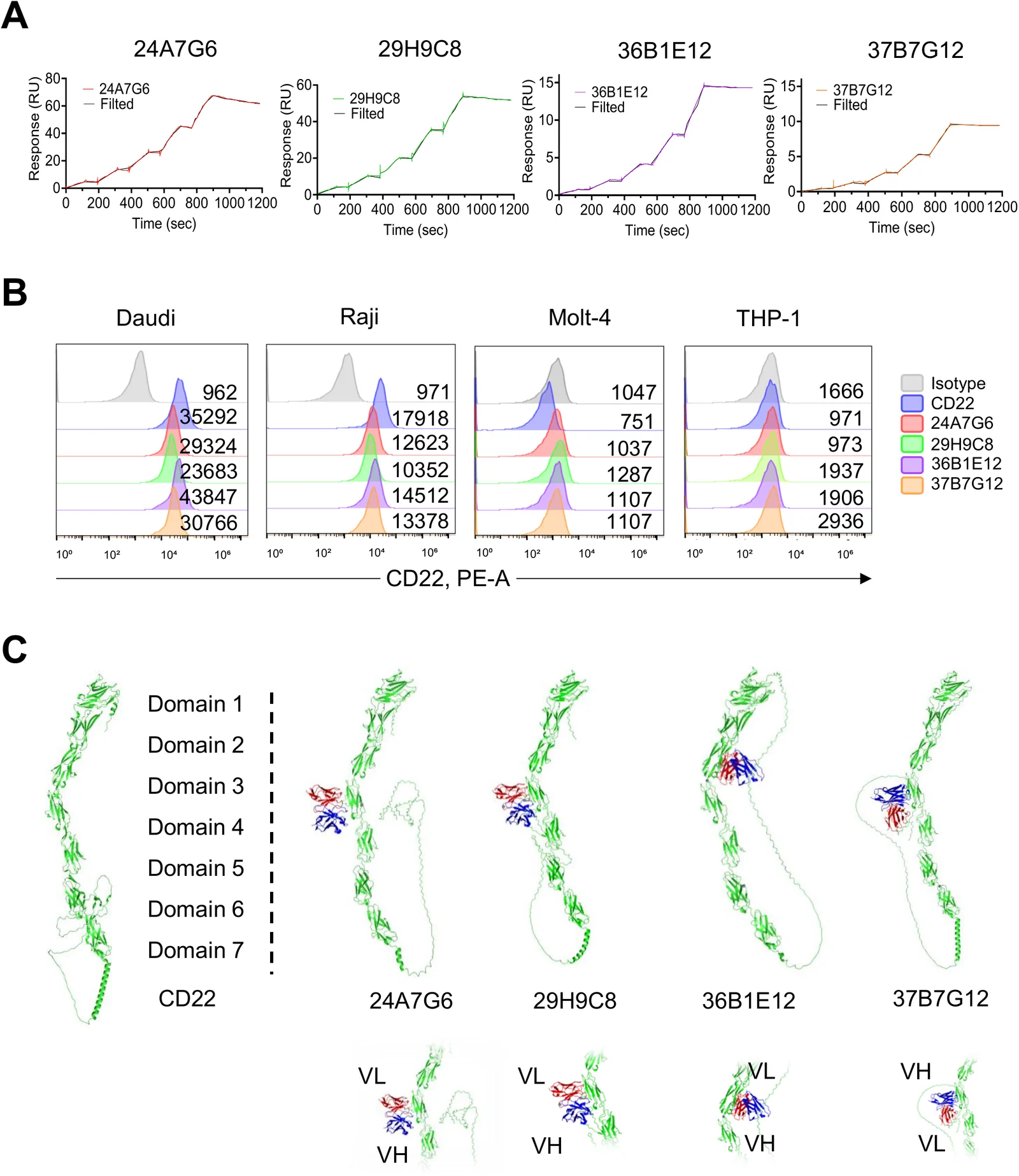
Characterization of anti-CD22 monoclonal antibody clones. (A) Surface plasmon resonance (SPR) sensorgrams showing the binding kinetics of recombinant CD22-His protein to immobilized anti-CD22 monoclonal antibody clones (24A7G6, 29H9C8, 36B1E12, and 37B7G12) coated at 0.5 µg/mL. CD22 analytes were injected at concentrations of 1.645, 3.290, 6.580, 13.16, and 26.23 nM. Distinct association and dissociation profiles indicate differences in binding kinetics and affinities among the clones. (B) Flow cytometry analysis of antibody binding specificity to human cell lines expressing various levels of CD22. Daudi and Raji (CD22 ⁺ B-cell lines), Molt-4 (T-cell line), and THP-1 (monocytic line) were stained with each anti-CD22 clone or isotype control. Representative histograms are shown, with mean fluorescence intensity (MFI) values indicating CD22-binding strength. (C) Structural modeling of CD22–antibody interactions predicted by AlphaFold. The seven extracellular immunoglobulin-like domains of CD22 (D1–D7) are illustrated (left). Predicted domain-binding sites of each anti-CD22 clone (24A7G6, 29H9C8, 36B1E12, and 37B7G12) are depicted in complex with their variable heavy (VH) and variable light (VL) chain regions (right).

Binding kinetics of anti-CD22 antibody clones determined by surface plasmon resonance (SPR). The equilibrium dissociation constant (K_D) was calculated from the association (K_a) and dissociation (K_d) rates. All clones demonstrated high-affinity binding in the nanomolar range, with 36B1E12 and 37B7G12 showing the strongest affinities.

To explore potential epitope recognition, AlphaFold modeling was used to predict antibody–antigen interactions within the extracellular domain of CD22 (Fig 1C). Clones 24A7G6, 29H9C8, and 37B7G12 were predicted to interact primarily with domain 4, while 36B1E12 was predicted to target domain 3. Structural modeling of the variable heavy (VH) and light (VL) chain orientations indicated diverse paratope conformations, potentially accounting for the differences in antigen-binding kinetics. Together, these findings confirm the successful generation of high-affinity, CD22-specific mAbs with distinct binding properties, supporting their potential for downstream CAR construct development.

### Development and expression of anti-CD22 CAR constructs

We first generated CAR constructs using the variable regions from four newly developed anti-CD22 monoclonal antibody clones, incorporating a Strep-Tag II as a transduction marker together with an IgG2/CH3 hinge, CD28 transmembrane (TM) domain, and intracellular signaling domains (4−1BB and CD3ζ) (Fig 2A). Following transduction into primary human T cells, CAR expression was assessed by flow cytometry. Among the four clones, only 24A7G6 and 29H9C8 demonstrated efficient surface CAR expression, with more than 80% of cells positive for both Strep-Tag II and CD22 binding—showing extremely high statistical significance compared with 36B1E12 and 37B7G12 (p < 0.0001) (Fig 2B). Based on these results, these two clones were selected for further characterization. To evaluate the effect of costimulatory domains on CAR expression and stability, second-generation constructs containing either 4−1BB or CD28 intracellular signaling domains were generated (Fig 2C). Transduction efficiency and CAR expression levels were analyzed on days 4 and 11 post-transduction (Fig 2D). T cells transduced with 4–1BB-based CARs maintained higher and more stable expression over time compared with CD28-based CARs (p < 0.05) (Fig 2E). Specifically, CAR expression in 4−1BB constructs remained above 70%, whereas CD28 constructs showed a decline by day 11. However, geometric mean fluorescence intensity (gMFI) did not differ significantly among the constructs at either day 4 or day 11 (Fig 2F), suggesting that the level of CAR expression per CAR-positive cell was comparable across groups.These findings indicate that the choice of costimulatory domain influences the persistence of CAR expression in transduced T cells.

**Fig 2:**
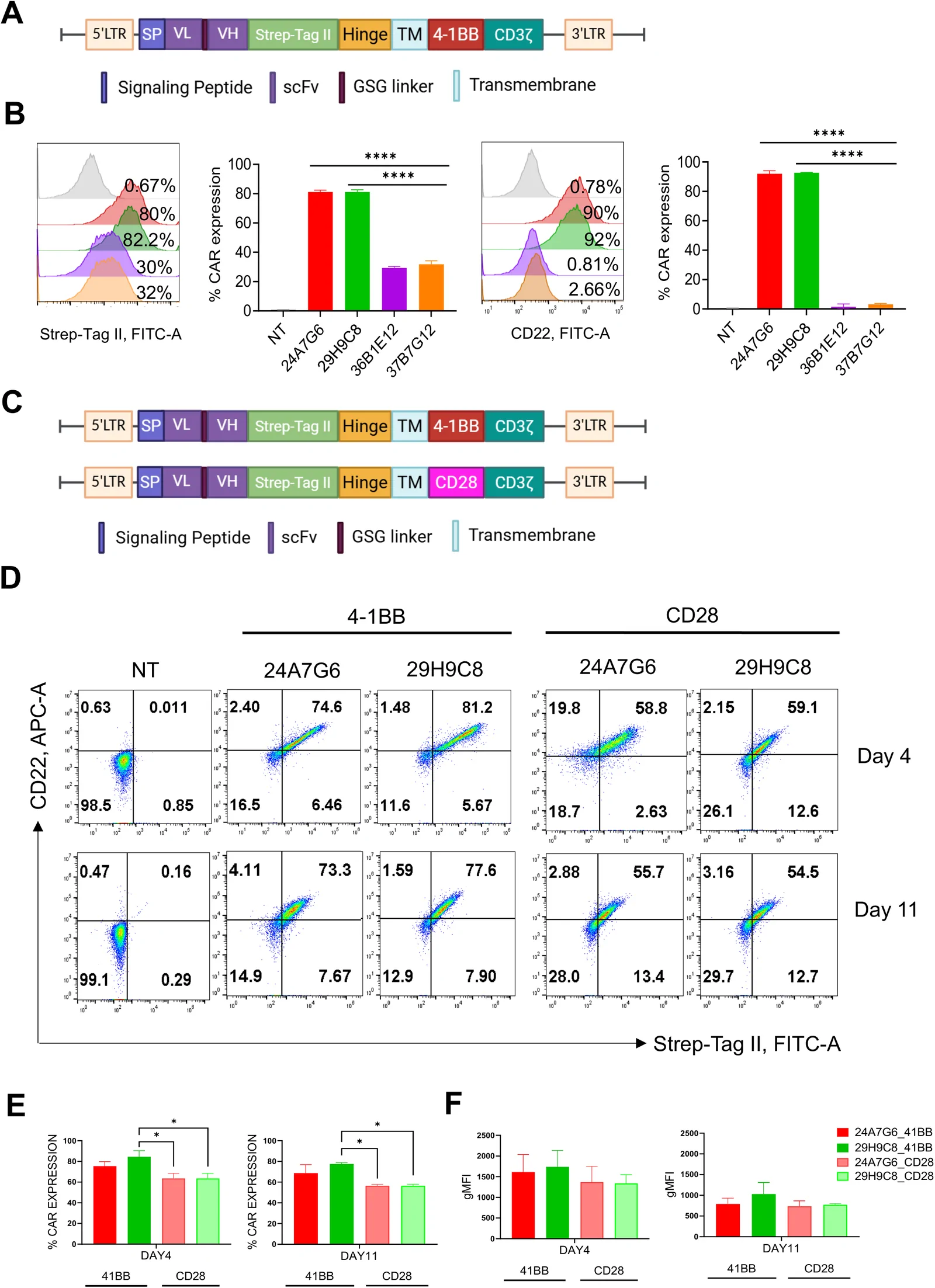
Development and expression of anti-CD22 CAR constructs. (A) Schematic representation of second-generation CAR constructs derived from anti-CD22 clones (24A7G6, 29H9C8, 36B1E12, and 37B7G12). Each construct includes the scFv region, a Strep-Tag II for detection, a hinge and transmembrane (TM) domain, followed by the 4−1BB costimulatory domain and the CD3ζ signaling domain. (B) Flow cytometry analysis of CAR expression in transduced T cells. Representative histograms and quantification graphs show staining with Strep-Tag II and CD22 compared with non-transduced (NT) controls, confirming efficient surface expression of CAR molecules. (C) Alternative CAR constructs incorporating either 4−1BB or CD28 costimulatory domains (shown for 24A7G6 and 29H9C8) retain the same Strep-Tag II, hinge, TM, and CD3ζ domains. (D) Flow cytometric analysis of CAR expression stability at days 4 and 11 post-transduction. Representative dot plots display dual staining with CD22 (APC-A) and Strep-Tag II (FITC-A) for NT and CAR T cells generated from 24A7G6 and 29H9C8 clones. Quantification of double-positive cells (bottom panels) demonstrates sustained CAR expression in both 4−1BB and CD28 constructs over time. (E) Quantification of CAR-positive T cells on days 4 and 11 for each construct. (F) Quantification of geometric mean fluorescence intensity (gMFI) of CAR-positive T cells measured by Strep-Tag II on days 4 and 11 for each construct. Data are presented as mean ± SEM from three independent experiments. Statistical significance was determined by one-way ANOVA (*p < 0.05, ***p < 0.0001).

### Expansion and phenotypic characterization of anti-CD22 CAR T Cells

The proliferative capacity of CAR T cells was monitored for 11 days post-transduction. Both 24A7G6- and 29H9C8-derived CAR T cells containing either 4−1BB or CD28 costimulatory domains expanded efficiently, showing growth kinetics comparable to non-transduced (NT) T cells (Fig 3A). Analysis of T-cell subsets revealed a predominance of CD8⁺ over CD4 ⁺ cells, maintaining a consistent ratio of approximately 60–70% to 30–40% across all conditions (Fig 3B). Phenotypic profiling showed that most CD4⁺ and CD8 ⁺ T cells retained a naïve phenotype (T_N, ~ 50–65%), with notable representation of central memory (T_CM, ~ 15–25%) and effector memory (T_EM, ~ 10–20%) subsets compared with NT controls. Effector T cells (T_E) were observed at lower frequencies (~10–15%), particularly within the CD8 ⁺ population (Fig 3C). These results suggest that CAR transduction does not impair proliferation and promotes the maintenance of memory-associated phenotypes supportive of long-term persistence.

**Fig 3:**
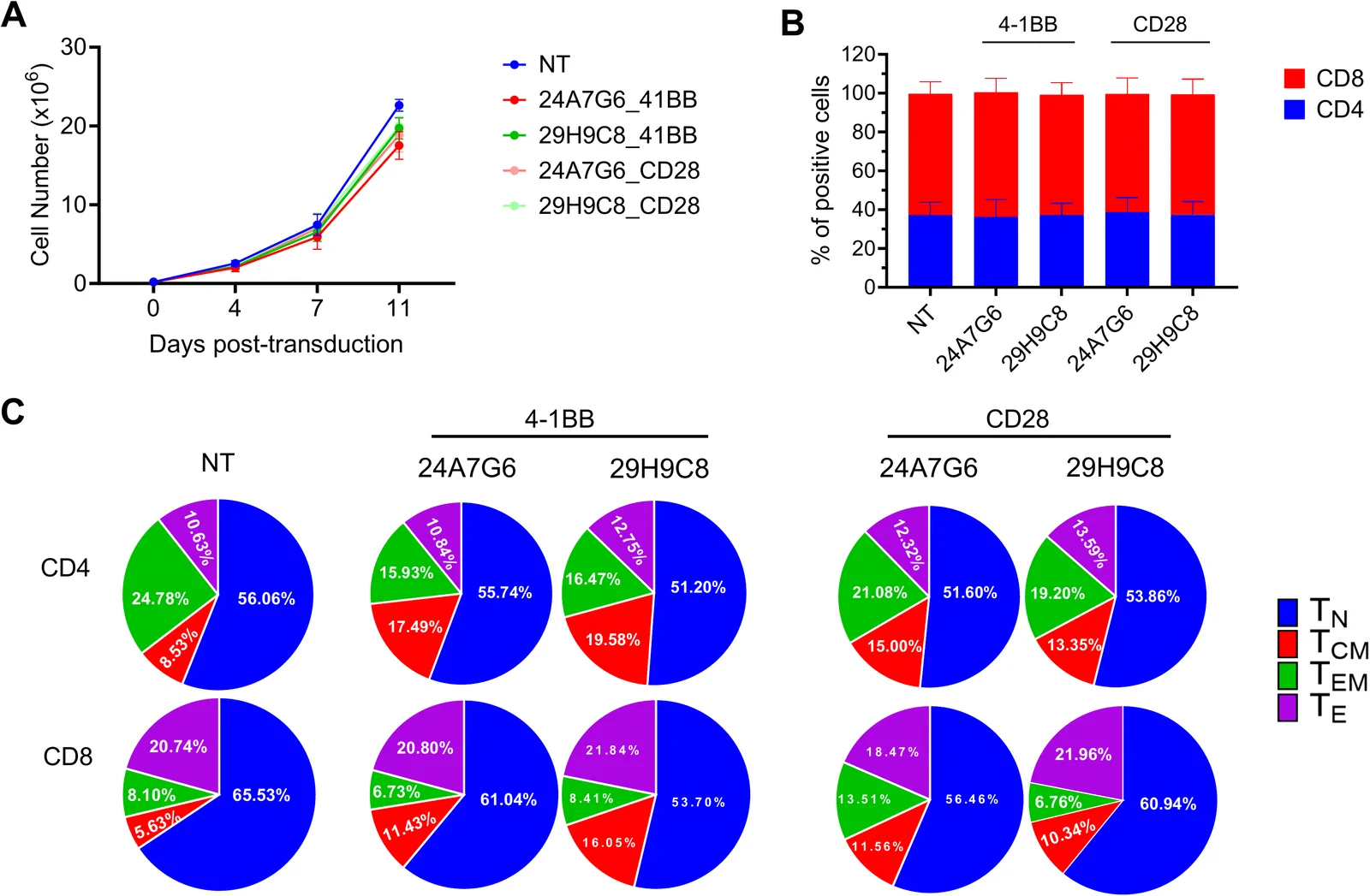
Expansion kinetics, lineage distribution, and memory phenotype of CAR-transduced T cells. T cells were transduced with CARs bearing scFvs 24A7G6 or 29H9C8 and either 4−1BB or CD28 costimulatory domains; NT T cells served as controls. Unless indicated, analyses were performed on day 11 post-transduction.(A) Cell expansion on days 0, 4, 7, and 11. Total viable cells were enumerated by trypan-blue exclusion. Lines show mean ± SEM of counts (×10⁶) for each group (4−1BB, CD28, NT). (B) Lineage distribution on day 11. Bars depict the percentage of CD4⁺ and CD8 ⁺ cells within each group (mean ± SEM). (C) Memory phenotype of CD4⁺ and CD8 ⁺ subsets. Pie charts show naïve (T_N), central memory (T_CM), effector memory (T_EM), and terminal effector (T_E) fractions; values indicate the percentage within the total CD4⁺ or CD8 ⁺ pool for each group. Data are mean ± SEM from four independent experiments.

### Cytotoxic activity of CAR T cells against CD22⁺  target cells

We evaluated the antigen-specific cytotoxicity of anti-CD22 CAR T cells against CD22⁺ (Raji and Nalm6) and CD22⁻ (K562) target cells using an in vitro killing assay at various effector-to-target (E:T) ratios over 24, 48, and 72 hours (Fig 4). Both 4−1BB– and CD28-based CAR T cells exhibited clear time- and dose-dependent cytotoxicity against CD22 ⁺ targets. For 4−1BB–containing constructs, 24A7G6-4–1BB and 29H9C8-4–1BB T cells demonstrated progressively increased killing, achieving approximately 60–80% lysis of Nalm6 cells and 40–60% lysis of Raji cells at the highest E:T ratio (1:1) after 72 hours. CD28-based CAR T cells (24A7G6-CD28 and 29H9C8-CD28) showed a similar trend, with reduced but still measurable activity at lower E:T ratios down to 1:4. Non-transduced (NT) T cells exhibited minimal cytotoxicity (<10%) under all conditions and no activity against CD22 ⁻ K562 cells, confirming the antigen specificity of the CAR constructs. Although 29H9C8-derived CARs tended to display slightly higher cytotoxic activity than 24A7G6-derived CARs, this difference was not statistically significant.

**Fig 4:**
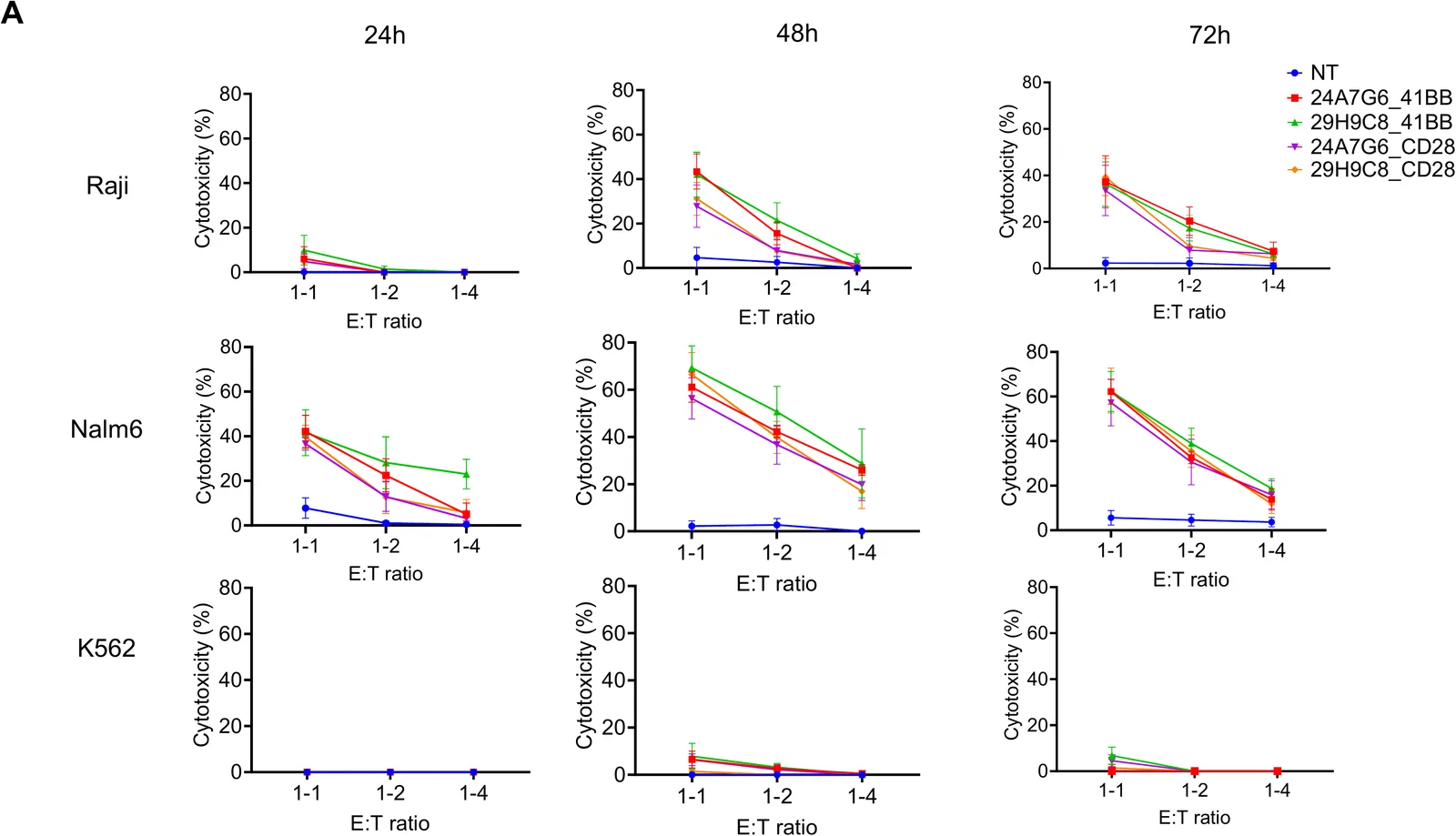
Cytotoxicity of CAR T cells containing 4-1BB or CD28 costimulatory domains. CAR T cells expressing 24A7G6 or 29H9C8 scFvs paired with either 4-1BB or CD28 were co-cultured with CD22 ⁺ Raji (top) and Nalm-6 (middle), or CD22 ⁻ K562 (bottom) targets at effector-to-target (E:T) ratios of 1:1, 1:2, and 1:4. Target-cell lysis was assessed at 24, 48, and 72 h. NT T cells served as negative controls. CAR T cells exhibited time- and dose-dependent cytotoxicity against CD22 ⁺ targets, with negligible killing of CD22 ⁻ K562, confirming antigen specificity. Data are mean ± SEM from four independent experiments.

To further assess CAR T-cell functionality following antigen engagement, we analyzed the expression of exhaustion and activation markers (S1 Fig). Baseline levels of TIGIT, PD-1, LAG-3, and TIM-3 were below 10% prior to antigen stimulation. After co-culture with CD22 ⁺ target cells, these exhaustion markers increased to approximately 15–25%, with significant elevations in PD-1, LAG-3, and TIM-3 (*p* < 0.05 or *p* < 0.01), particularly in CD28-based CAR T cells. TIGIT expression also increased moderately across both costimulatory domains. Activation markers remained consistently expressed, with CD25 detected in more than 80% of cells both before and after stimulation, and CD69 maintained at 20–40% without substantial variation among groups. Overall, both 4−1BB– and CD28-based CAR T cells demonstrated potent, antigen-specific, time- and dose-dependent cytotoxicity against CD22 ⁺ targets, achieving up to ~80% lysis at higher E:T ratios by 72 hours, with 24A7G6- and 29H9C8-derived CAR T cells exhibiting comparable functional activity.

### Cytokine secretion profile of CAR T cells following target cell stimulation

To further assess the functional activity of the CAR T cells, cytokine production was analyzed following antigen stimulation (Fig 5). Compared with non-transduced (NT) T cells, all CAR T-cell constructs exhibited elevated cytokine secretion. Among the tested cytokines, IFN-γ showed the most pronounced increase, rising from approximately 15–20 pg/mL in NT T cells to 40–60 pg/mL in 24A7G6-derived CAR T cells and 50–70 pg/mL in 29H9C8-derived CAR T cells, representing a statistically significant increase compared with NT controls (*p* < 0.05). TNF levels were also significantly elevated, increasing from approximately 5 pg/mL in NT T cells to 15–30 pg/mL in CAR T cells (*p* < 0.05, *p < 0.01). IL-2 secretion showed only a slight, non-significant increase across CAR T-cell groups.

**Fig 5:**
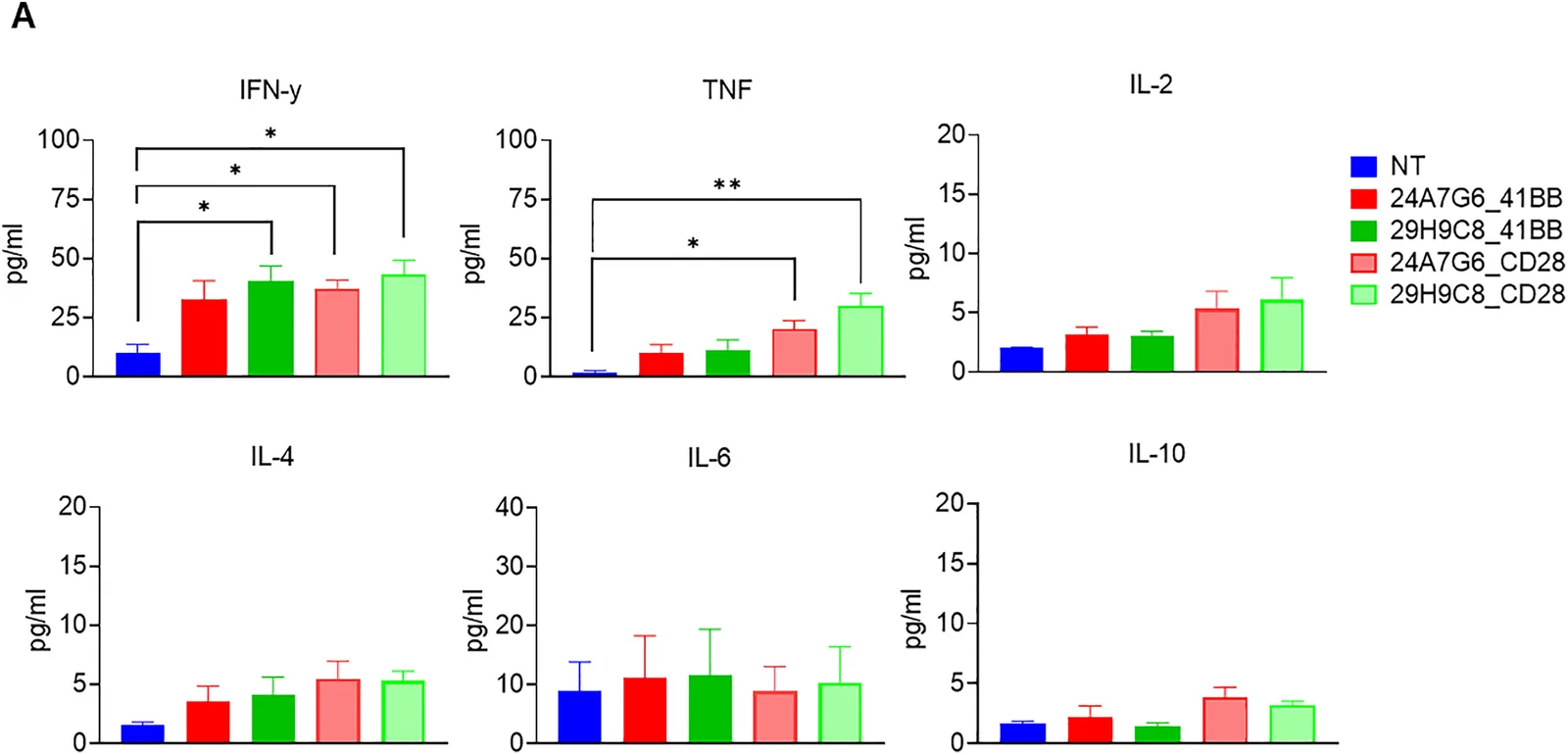
Cytokine secretion profile of anti-CD22 CAR T cells. (A) Supernatants collected from non-transduced T cells (NT) and CAR T cells expressing anti-CD22 scFvs (24A7G6 or 29H9C8) with either 4−1BB or CD28 costimulatory domains were analyzed for cytokine production. Concentrations of IFN-γ, TNF, IL-10, IL-6, IL-4, and IL-2 were quantified using the BD™ Cytometric Bead Array (CBA) Human Inflammatory Cytokine Kit. Data are presented as mean ± SEM from three independent experiments. Statistical significance was determined by one-way ANOVA (*p < 0.05, *p < 0.01).

In contrast, cytokines associated with Th2 or regulatory responses (IL-4, IL-6, and IL-10) were detected at relatively low concentrations (<10 pg/mL) and showed no significant differences among groups. Overall, the cytokine profile was dominated by type 1 effector cytokines, particularly IFN-γ and TNF, indicating robust CAR-mediated T-cell activation without excessive inflammatory or immunosuppressive cytokine production. These findings suggest that both 24A7G6- and 29H9C8-derived CAR T cells are functionally competent upon CD22 antigen engagement, producing characteristic effector cytokines associated with T-cell activation.

### Long-term anti-tumor activity and persistence of anti-CD22 CAR-T cells

To evaluate the durability of anti-tumor responses and the persistence of CAR-T cells under chronic antigen exposure, we performed long-term co-culture assays and repetitive antigen challenge experiments. In the long-term killing assay, CAR-T cells were co-cultured with Raji cells at a low E:T ratio (1:10) and monitored on day 1, 4, 7, and 12 (Fig 6A). The anti-tumor activity of CAR-T was found at day 4, when the residual target cells of all CAR-T groups obviously decreased. In contrast, the target only and NT groups showed rapid and uncontrolled proliferation. All anti-CD22 CAR-T constructs effectively suppressed tumor growth, especially the 24A7G6-CD28, which maintained the lowest levels of residual target cells through day 12, the result was significantly different when compared with 24A7G6-4–1BB CAR (p < 0.05) (Fig 6B).

**Fig 6:**
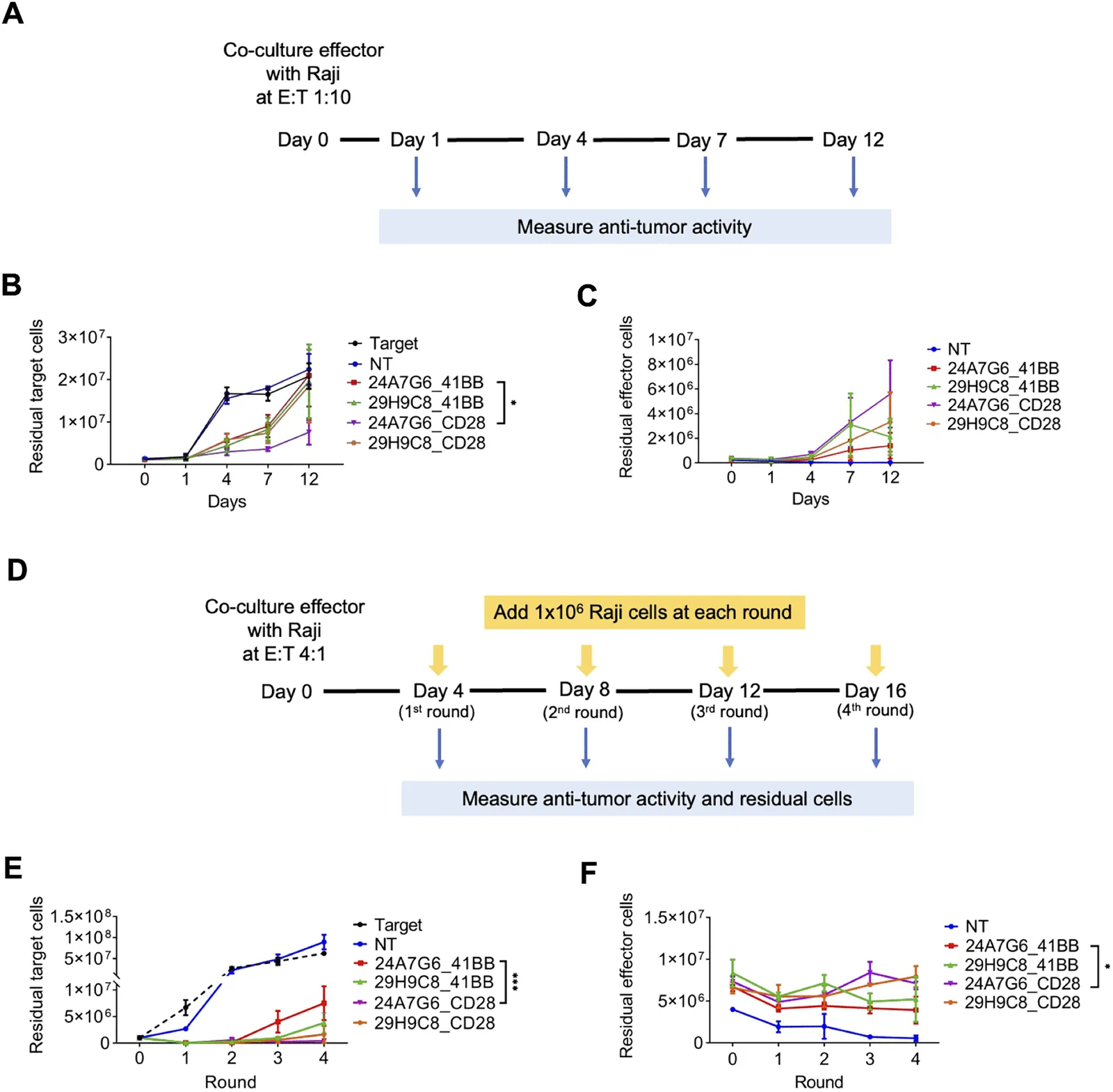
Evaluation of long-term cytotoxicity and sequential co-culture rechallenge of anti-CD22 CAR-T cells. (A) Schematic diagram of the long-term cytotoxicity assay. Effector cells (NT or CAR-T cells) were co-cultured with Raji cells at a low E:T ratio of 1:10. Anti-tumor activity and residual cell counts were monitored on days 1, 4, 7, and 12.(B) Quantification of residual target cells and (C) effector cells in the long-term co-culture assay were monitored alongside the elimination of target cells. (D) Experimental schematic of the sequential co-culture rechallenges. The effector cells were initially co-cultured with Raji cells at E:T ratio of 4:1. One million Raji cells were added sequentially every 4 days for a total of 4 rounds. Anti-tumor activity and residual cells were measured at each time point. (E) The residual target cells and (F) effector cells numbers were measured during the sequential co-culture rechallenge. Data are represented as mean ± SEM from three independent donors (n = 3). Statistical analysis was determined using two-way ANOVA with Tukey's multiple comparisons test (* p < 0.05, *** p < 0.001).

While the number of residual effector cells of all CAR-T remained low as the NT control until day 7, the CAR-T groups exhibited various levels of T cell persistence. Although the residual number of 24A7G6-CD28 CAR showed a higher number than the other CAR constructs by the end of the assay, this increase did not reach statistical significance (Fig 6C).

To investigate the exhaustion and proliferative capacity of the CAR-T cells under more rigorous conditions, a tumor cell rechallenge assay was assessed. Effector cells were rechallenged 1 x 10^6^ Raji cells every four days for four consecutive rounds at an initial E:T ratio of 4:1 (Figure 6D). All anti-CD22 CAR-T constructs demonstrated robust and sustained anti-tumor activity, maintaining residual target cell counts lower than those of the NT and tumor-only controls (Fig 6E). The 24A7G6-CD28 CAR provided significantly lower residual target cells than the 24A7G6-4–1BB CAR at round 4 (p < 0.001), while this potency was not revealed in clone 29H9C8.

During the tumor cells rechallenges, the CAR-T cells successfully expanded and persisted, with residual effector cell number remaining stable over round 4, whereas NT cells exhibited a progressive decline in cell number (Fig 6F). Interestingly, the 24A7G6-CD28 CAR showed significantly higher residual effector number at round 3 compared with the 24A7G6-4–1BB CAR (p < 0.05).

These results indicate that the CD28 co-stimulatory domain supported the long-term anti-tumor efficacy and sustained proliferation, especially 24A7G6-derived CAR-T cells.

## Discussion

In this study, we generated and characterized four anti-CD22 monoclonal antibody clones—24A7G6, 29H9C8, 36B1E12, and 37B7G12—using hybridoma technology. Each clone exhibited distinct binding kinetics and expression characteristics. Surface plasmon resonance and domain-binding analyses revealed that 24A7G6 and 29H9C8 displayed high specificity and well-defined kinetic profiles, whereas 36B1E12 and 37B7G12 showed slower binding or reduced CAR surface expression. These findings underscore the importance of pre-screening antibody candidates not only for affinity but also for expression efficiency prior to CAR integration, as scFv quality critically affects downstream CAR functionality [9,10,13,22].

Using 24A7G6- and 29H9C8-derived scFvs, we generated CAR constructs containing either 4−1BB or CD28 co-stimulatory domains and evaluated their in vitro function. Both scFv-derived CAR T-cell products demonstrated potent and antigen-specific cytotoxicity against CD22-positive target cells, with negligible activity against CD22-negative K562 cells, confirming target-dependent killing. In the short-term assays, cytotoxicity was time- and dose-dependent across all constructs, and no clear differences were observed between the 24A7G6- and 29H9C8-derived CAR T cells. These findings indicate that both newly generated scFvs are functionally competent recognition domains for CD22-targeted CAR T-cell design.

The initial 72-hour cytotoxicity assay suggested broadly comparable short-term activity between 4−1BB- and CD28-based CAR constructs. This observation is consistent with prior reports showing that both co-stimulatory domains can effectively support CAR T-cell activation, although through distinct signaling mechanisms. CD28 signaling is generally associated with rapid effector activation, glycolytic metabolism, and early cytokine production, whereas 4−1BB signaling has been linked to enhanced mitochondrial fitness, survival, and long-term persistence [22–32]. However, because such differences may not become apparent during short observation periods, we extended the functional evaluation by performing long-term co-culture and sequential tumor rechallenge assays under cytokine-free conditions.

These additional experiments provided a more stringent assessment of sustained antitumor activity and effector-cell persistence under chronic or repetitive antigen exposure. All anti-CD22 CAR T-cell constructs retained durable tumor control compared with non-transduced T cells, supporting the capacity of both 24A7G6- and 29H9C8-derived CAR T cells to maintain functional activity beyond the initial short-term assay window. Importantly, the longer-term studies also revealed differences that were not detected in the 72-hour assay. In particular, the 24A7G6-CD28 CAR T cells showed lower residual tumor-cell numbers than the corresponding 24A7G6-4–1BB construct in the long-term co-culture assay and at the fourth round of tumor rechallenge. In addition, 24A7G6-CD28 CAR T cells showed higher residual effector-cell numbers at an intermediate rechallenge time point. By contrast, this distinction between CD28 and 4−1BB was not clearly observed in the 29H9C8-derived CAR constructs. Taken together, these findings suggest that the functional impact of co-stimulatory domain choice may be context dependent and influenced by the specific scFv framework, and that differences between CD28 and 4−1BB signaling may emerge more clearly only under prolonged antigen stress.

To further assess functional activation, immune checkpoint expression was analyzed following antigen encounter (S2 Fig). Baseline levels of TIGIT, PD-1, LAG-3, and TIM-3 were low across all constructs. Upon co-culture with CD22 ⁺ target cells, significant upregulation of PD-1, LAG-3, and TIM-3 was observed, particularly in CD28-based CAR T cells, with TIM-3 showing the most prominent increase (~15–20%) across both costimulatory backbones. Transient upregulation of these markers, especially TIM-3, has been associated with activation rather than exhaustion during acute stimulation [33,34]. The limited PD-1 and LAG-3 expression (<15%) suggests that the tested conditions did not induce substantial exhaustion, consistent with previous findings that appropriately tuned antigen engagement can activate CAR T cells without driving terminal exhaustion [35].

Cytokine profiling revealed a balanced Th1-dominant response in both 24A7G6- and 29H9C8-derived CAR T cells, characterized by strong IFN-γ secretion and pronounced TNF induction. IL-2 production showed only a modest, non-significant increase, while IL-4, IL-6, and IL-10 levels remained minimal. This cytokine pattern reflects effective antigen-driven activation without excessive inflammatory signaling, thereby reducing the likelihood of cytokine release syndrome while maintaining robust effector function [36]. Notably, the comparable cytokine output between 4−1BB and CD28 constructs reinforces that both costimulatory domains can sustain similar early activation thresholds under short-term stimulation [37–39].

The absence of cytotoxicity toward CD22 ⁻ K562 cells reinforces the antigen specificity and safety of these CAR constructs, an essential consideration for minimizing on-target/off-tumor toxicity [40]. Structural modeling predicted that both 24A7G6 and 29H9C8 recognize domain 4 of CD22, which may explain the similar cytotoxic and activation profiles of their respective CAR T cells [9,13]. Systematic characterization at the antibody level, as demonstrated here, enables rational selection of scFv candidates that balance affinity, specificity, and expression efficiency—key factors for developing safe and effective CARs [41].

Several limitations of this study should be acknowledged. First, a clinically validated CD22-directed CAR, such as an m971-based construct, was not included as a benchmark. Therefore, although the newly generated CAR T cells demonstrated antigen-specific in vitro activity, the present data do not establish their relative performance compared with established CD22 CAR platforms. Second, detailed immunophenotypic characterization was performed at a selected evaluation time point rather than longitudinally throughout culture, limiting assessment of temporal changes in T-cell differentiation and memory phenotype. Third, functional studies were performed using established CD22-positive cell lines rather than primary patient-derived leukemia or lymphoma samples. Although these standardized models permit reproducible comparison among CAR constructs, they do not fully recapitulate the biological heterogeneity, antigen-density variation, and treatment susceptibility of primary malignancies. Finally, in vivo efficacy and safety were not evaluated. The long-term co-culture and sequential tumor-rechallenge assays provide evidence of sustained in vitro activity under prolonged antigen exposure but cannot assess CAR T-cell trafficking, expansion, persistence, tumor control in vivo, or potential toxicity. Accordingly, the present findings should be interpreted as an initial in vitro characterization that supports prioritization of candidate CAR constructs for further preclinical development. Future studies incorporating established CAR comparators, longitudinal phenotypic assessment, patient-derived tumor samples, and in vivo efficacy and safety evaluation will be required to establish the translational potential of the lead constructs.

## Conclusion

The present study identified novel anti-CD22 monoclonal antibodies generated by immunization with recombinant CD22 protein. Among the antibody clones obtained, 24A7G6 and 29H9C8 were selected as lead candidates based on their high-affinity CD22 binding and favorable CAR surface expression. Structural modeling predicted that both antibodies recognize domain 4 of the CD22 extracellular region. Importantly, scFvs derived from these two antibodies retained functional antigen recognition when incorporated into second-generation CAR constructs and mediated specific in vitro activity against CD22-positive target cells. Among the tested constructs, the 24A7G6-CD28 CAR demonstrated more sustained antitumor activity during prolonged co-culture and repeated tumor-rechallenge assays. These findings support 24A7G6 and 29H9C8 as promising new antibody-derived targeting domains for CD22-directed CAR T-cell development. Further in vivo characterization of efficacy, persistence, and safety is warranted to determine their translational potential.

## Supporting information

S1 Fig Flow cytometry gating strategies for CAR T-cell characterization and functional assays. Representative gating strategies used in this study. (A) Transduction efficiency was determined by gating singlets and viable cells followed by identification of CAR-positive cells using Strep-Tag II and/or CD22 staining. (B) T-cell subsets were identified by gating viable CD3 ⁺ cells and then separating CD4⁺ and CD8 ⁺ populations. (C) Memory phenotype was analyzed within viable CD3⁺ or CD4 ⁺ /CD8 ⁺ T-cell populations using CD45RO and CCR7 to define naïve (T_N), central memory (T_CM), effector memory (T_EM), and terminal effector (T_E) subsets. (D) Exhaustion markers were assessed in viable CD3 ⁺ T cells by analysis of TIGIT, PD-1, TIM-3, and LAG-3 expression. (E) Activation markers were assessed in viable CD3 ⁺ T cells by analysis of CD25 and CD69 expression. (F) Cytotoxicity was evaluated by gating viable effector T cells (7-AAD ⁻ CD3⁺) and viable target tumor cells (7-AAD ⁻ GFP⁺) to quantify residual cell populations after co-culture.
(TIF)

S2 Fig Expression of inhibitory and activation markers in newly generated anti-CD22 CAR T cells. (A) Flow-cytometric analysis of inhibitory receptors TIGIT, PD-1, LAG-3, and TIM-3 on CAR T cells engineered with either CD28 or 4−1BB costimulatory domains. Cells were assessed before and after stimulation with CD22 ⁺ target cells. Anti-CD22 scFvs 24A7G6 and 29H9C8 were compared with non-transduced (NT) T cells. (B) Expression of activation markers CD25 and CD69 was measured under the same conditions. Data are presented as mean ± SEM from three independent experiments. Statistical significance was determined by one-way ANOVA with Tukey’s post hoc test (*p < 0.05, *p < 0.01).
(TIF)

S3 Dataset Minimal dataset underlying the findings reported in this study. The dataset contains the source data underlying the quantitative analyses presented in Figs 1–6 and S2 Fig, including anti-CD22 antibody-binding kinetics, CAR expression and transduction efficiency, T-cell expansion and phenotypic characterization, cytotoxicity assays, cytokine secretion, activation and inhibitory-marker expression, long-term co-culture assays, and sequential tumor-rechallenge experiments. Replicate-level values and corresponding summary data are provided where applicable.
(PDF)
