# Supplementary material for: Generation and in vitro functional characterization of anti-CD22 chimeric antigen receptor T cells derived from newly generated monoclonal antibody clones
Source: PLoS One. 2026 Nov 12;21(11):e0362221. doi: 10.1371/journal.pone.0362221 (PMC13759115; doi:10.1371/journal.pone.0362221)

Data set: Generation and In Vitro Functional Characterization of Anti-CD22 Chimeric Antigen Receptor T Cells Derived from Newly Generated Monoclonal Antibody Clones

Figure 1

A. KINETIC BINGDING CD22

Clone	KD(M)
24A7G6	**1.086E-9**
29H9C8	**6.370E-10**
36B1E12	**5.563E-10**
37B7G12	**5.006E-10**

Sample: mAb clone 24A7G6

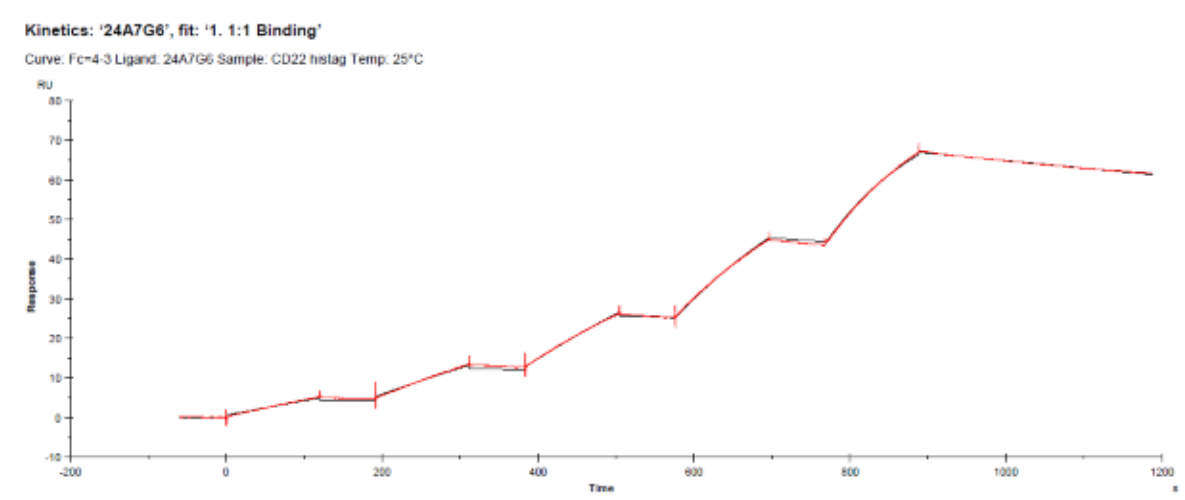

Ligand	Analyte	Chi2	Ka(1/Ms)	Kd(1/s)	KD(M)	Rmax
mAb:24A7G6	CD22 histag	0.126	2.706E+5	2.940E-4	1.086E-9	86.20

Sample: mAb clone 29H9C8

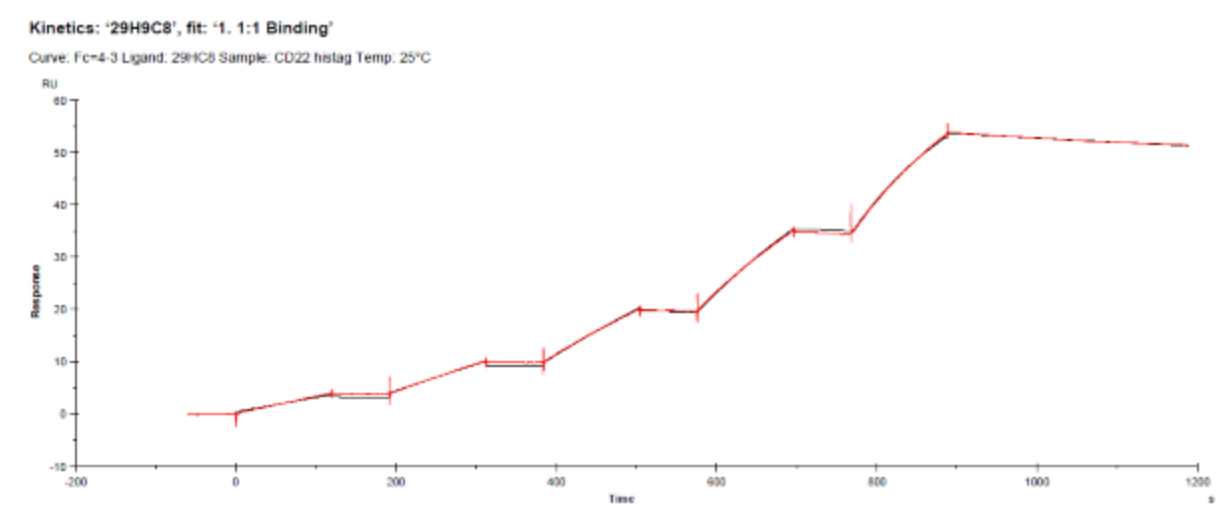

Ligand	Analyte	Chi2	Ka(1/Ms)	Kd(1/s)	KD(M)	Rmax
mAb:29H9C8	CD22 histag	0.104	2.419E+5	1.541E-4	6.370E-10	71.09

Sample: mAb clone 36B1E12

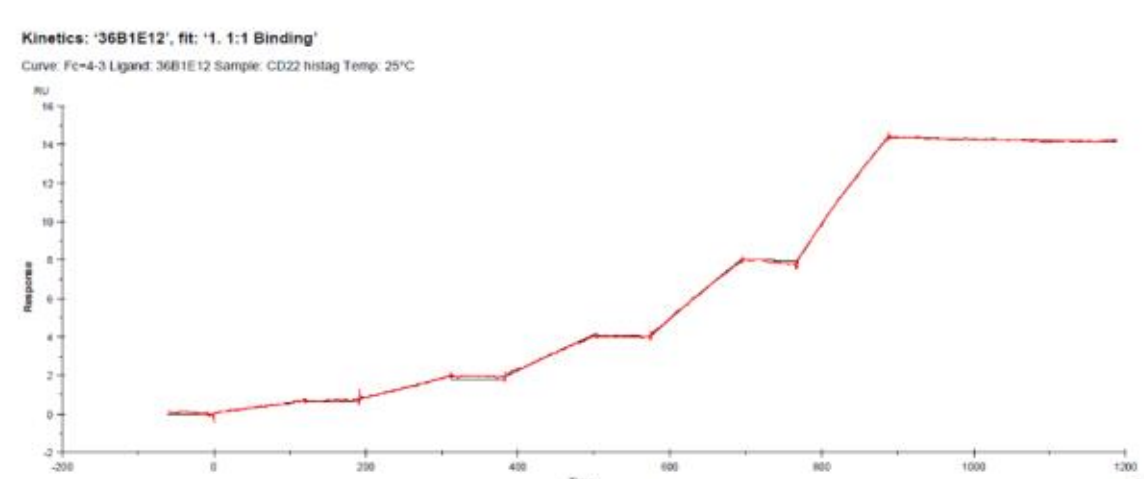

Ligand	Analyte	Chi2	Ka(1/Ms)	Kd(1/s)	KD(M)	Rmax
mAb:36B1E12	CD22 histag	0.00460	9.590E+4	5.335E-5	5.563E-10	32.71

Sample: mAb clone 37B7G12

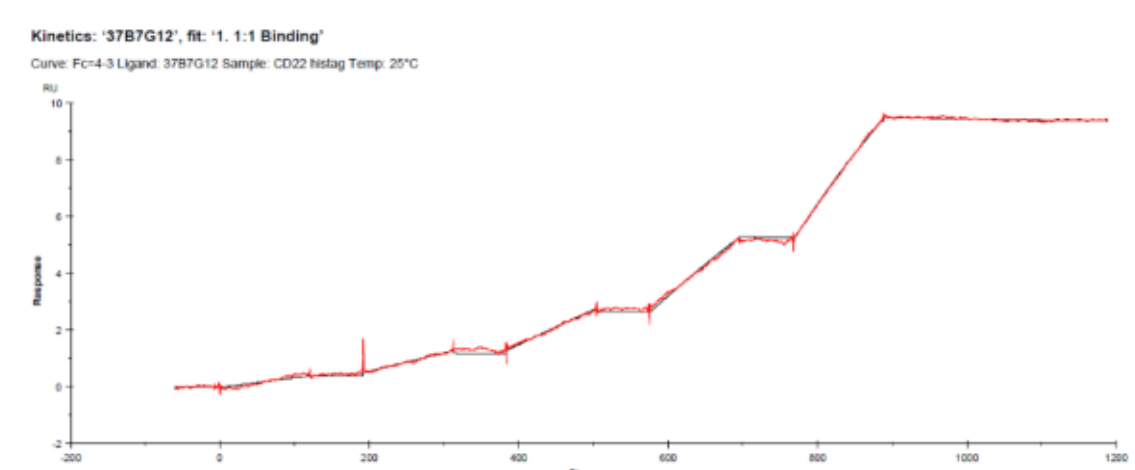

Ligand	Analyte	Chi2	Ka(1/Ms)	Kd(1/s)	KD(M)	Rmax
mAb:37B7G12	CD22 histag	0.00580	9.391E+4	4.701E-5	5.006E-10	21.94

Figure 2

B. EXPRESSION (%)

Group	scFv clone	Replicate			Mean
		N1	N2	N3	
Strep-Tag II	NT	0.87	0.67	0.76	0.77
	24A7G6	80.20	82.00	81.00	81.07
	29H9C8	82.20	80.00	81.20	81.13
	36B1E12	30.00	28.60	29.40	29.33
	37B7G12	33.5	30.00	31.80	31.77
CD22 Ligand	NT	0.87	0.67	0.76	0.77
	24A7G6	90.50	93.5	92.00	92.00
	29H9C8	92.90	92.5	92.80	92.73
	36B1E12	0.08	2.88	1.50	1.49
	37B7G12	2.60	3.58	3.10	3.09

E. EFFICIENCY (Strep Tag II)(%)

Group	CAR construct	Replicate			Mean
		N1	N2	N3	
Day4	24A7G6_4-1BB	72.30	70.00	84.00	75.43
	29H9C8_4-1BB	74.60	84.00	95.00	84.53
	24A7G6_CD28	72.60	61.70	56.50	63.60
	29H9C8_CD28	69.90	66.60	56.30	64.27
Day11	24A7G6_4-1BB	53.10	73.30	80.00	68.80
	29H9C8_4-1BB	77.60	75.00	80.00	77.53
	24A7G6_CD28	54.50	59.00	56.20	56.57
	29H9C8_CD28	53.30	58.30	57.00	56.20

F. gMFI (Strep-Tag II)

Group	CAR construct	Replicate			Mean
		N1	N2	N3	
Day4	24A7G6_4-1BB	1451	977	2414	1614.00
	29H9C8_4-1BB	1511	1191	2511	1737.67
	24A7G6_CD28	1274	764	2074	1370.67
	29H9C8_CD28	1223	1042	1751	1338.67
Day11	24A7G6_4-1BB	841	531	1001	791.00
	29H9C8_4-1BB	823	687	1586	1032.00
	24A7G6_CD28	849	474	880	734.33
	29H9C8_CD28	766	712	830	769.33

Figure 3

A. CELL EXPANSION ($x10^6$ cells)

Group	CAR construct	Replicate	Mean

		N1	N2	N3	N4	
Day0	NT	0.20	0.20	0.20	0.20	0.20
	24A7G6_4-1BB	0.20	0.20	0.20	0.20	0.20
	29H9C8_4-1BB	0.20	0.20	0.20	0.20	0.20
	24A7G6_CD28	0.20	0.20	0.20	0.20	0.20
	29H9C8_CD28	0.20	0.20	0.20	0.20	0.20
Day4	NT	2.62	1.64	2.50	3.44	2.55
	24A7G6_4-1BB	1.08	1.22	2.46	3.32	2.02
	29H9C8_4-1BB	1.56	1.72	2.54	2.88	2.18
	24A7G6_CD28	1.62	1.70	2.14	3.00	2.12
	29H9C8_CD28	1.66	1.22	2.14	3.54	2.14
Day7	NT	4.88	6.20	7.64	11.12	7.46
	24A7G6_4-1BB	4.56	4.92	3.64	10.48	5.90
	29H9C8_4-1BB	5.60	5.08	5.44	10.00	6.53
	24A7G6_CD28	6.12	4.28	5.68	12.00	7.02
	29H9C8_CD28	6.60	4.28	6.84	11.76	7.37
Day11	NT	22.48	21.60	24.80	21.60	22.62
	24A7G6_4-1BB	14.48	14.56	21.20	19.86	17.53
	29H9C8_4-1BB	15.68	21.20	21.44	20.48	19.70
	24A7G6_CD28	15.04	23.20	21.76	15.28	18.82
	29H9C8_CD28	18.56	23.28	18.19	19.76	19.95

B. T CELL SUBSET (%)

Group	CAR construct	Replicate				Mean
		N1	N2	N3	N4	
CD4	NT	45	29.3	43	34.4	37.93
	24A7G6_4-1BB	40	26.6	50	32.2	37.20
	29H9C8_4-1BB	39.3	26.4	40.7	39.5	36.48
	24A7G6_CD28	42	32	50.3	34.7	39.75
	29H9C8_CD28	42.2	31.5	47.1	33	38.45
CD8	NT	55	69.4	57	65.6	61.75
	24A7G6_4-1BB	60.1	71.7	54	67.8	63.40
	29H9C8_4-1BB	60	72.6	56.3	60.5	62.35
	24A7G6_CD28	58	68.1	47.6	65.3	59.75
	29H9C8_CD28	57.6	68.5	50.1	66.9	60.78

C. MEMORY PHYNOTYPE (%)

Group		CAR construct	Replicate				Mean
			N1	N2	N3	N4	
	T_N	NT	48.10	57.50	73.30	51.50	57.60
	T_{CM}		10.10	10.10	4.84	13.20	9.56
	T_{EM}		32.60	25.60	13.60	25.70	24.38
	T_E		9.13	6.79	8.22	9.69	8.46
	T_N	24A7G6_4-1BB	45.60	57.10	73.00	45.90	55.40
	T_{CM}		23.00	24.50	14.30	18.20	20.00
	T_{EM}		24.50	13.60	5.85	23.70	16.91
	T_E		6.96	4.87	6.79	12.30	7.73

CD4	T_N	29H9C8_4-1BB	40.80	54.00	71.20	44.50	52.63
	T_{CM}		30.10	24.00	15.40	14.20	20.93
	T_{EM}		20.00	16.20	6.44	26.80	17.36
	T_E		8.69	5.80	6.94	14.50	8.98
	T_N	24A7G6_CD28	47.30	54.50	62.60	43.40	51.95
	T_{CM}		18.10	19.50	13.00	16.40	16.75
	T_{EM}		27.80	20.70	10.70	28.40	21.90
	T_E		6.87	5.33	13.70	11.80	9.43
	T_N	29H9C8_CD28	45.20	56.90	64.50	44.00	52.65
	T_{CM}		20.00	17.20	12.40	13.20	15.70
	T_{EM}		27.20	20.80	13.20	26.20	21.85
	T_E		7.59	5.04	9.80	16.60	9.76
CD8	T_N	NT	68.10	57.00	68.40	79.70	68.30
	T_{CM}		12.40	8.84	3.51	0.79	6.39
	T_{EM}		8.98	8.42	13.80	3.02	8.56
	T_E		12.60	25.20	14.30	16.50	17.15
	T_N	24A7G6_4-1BB	52.70	48.30	69.50	81.10	62.90
	T_{CM}		24.80	11.90	11.70	4.08	13.12
	T_{EM}		10.10	7.15	8.59	2.38	7.06
	T_E		12.50	32.60	10.10	12.40	16.90
	T_N	29H9C8_4-1BB	39.00	47.10	62.40	78.70	56.80
	T_{CM}		37.10	13.40	13.20	4.23	16.98
	T_{EM}		11.00	9.43	11.00	2.48	8.48
	T_E		12.90	30.10	13.40	14.60	17.75
	T_N	24A7G6_CD28	47.30	54.50	62.60	43.40	51.95
	T_{CM}		18.10	19.50	13.00	16.40	16.75
	T_{EM}		27.80	20.70	10.70	28.40	21.90
	T_E		6.87	5.33	13.70	11.80	9.43
	T_N	29H9C8_CD28	51.80	42.00	69.30	76.60	59.93
	T_{CM}		28.50	9.58	8.36	2.61	12.26
	T_{EM}		8.57	9.74	10.50	3.83	8.16
	T_E		11.10	38.70	11.80	16.90	19.63

Figure 4

A. CYTOTOXIC (%)

Group			CAR construct	Replicate					Mean
				N1	N2	N3	N4	N5	
RAJI	24H	1-1	NT	0.00	0.00	0.00	0.00	0.00	0.00
		1-2		0.00	0.00	0.00	0.00	0.00	0.00
		1-4		0.00	0.00	0.00	0.00	0.00	0.00
		1-1	24A7G6_4-1BB	0.00	0.00	0.00	27.56	2.70	6.05
		1-2		0.00	0.00	0.00	0.00	0.00	0.00
		1-4		0.00	0.00	0.00	0.00	0.00	0.00
		1-1	29H9C8_4-1BB	0.00	0.00	16.38	33.17	0.00	9.91
		1-2		0.00	0.00	7.02	0.00	0.00	1.40
		1-4		0.00	0.00	0.00	0.00	0.00	0.00
		1-1	24A7G6_CD28	0.00	0.00	8.14	16.27	0.00	4.88
		1-2		0.00	0.00	0.00	0.00	0.00	0.00
		1-4		0.00	0.00	0.00	0.00	0.00	0.00
		1-1	29H9C8_CD28	0.00	0.00	0.00	23.47	0.00	4.69
		1-2		0.00	0.00	0.00	0.15	0.00	0.03
		1-4		0.00	0.00	0.00	0.00	0.00	0.00
	48H	1-1	NT	0.00	0.00	23.22	0.00	0.00	4.64

		1-2		0.00	0.00	12.77	0.00	0.00	2.55
		1-4		0.00	0.00	0.00	0.00	0.00	0.00
		1-1	24A7G6_4-1BB	54.80	64.31	19.88	31.68	46.38	43.41
		1-2		10.06	26.22	0.00	13.97	27.42	15.53
		1-4		0.00	0.00	0.00	0.00	0.00	0.00
		1-1	29H9C8_4-1BB	26.87	81.25	31.40	40.31	30.21	42.01
		1-2		0.00	48.70	23.62	16.14	18.60	21.41
		1-4		0.00	9.28	9.74	1.93	0.00	4.19
		1-1	24A7G6_CD28	25.47	12.50	16.27	19.69	64.80	27.75
		1-2		0.00	0.00	0.00	14.89	24.31	7.84
		1-4		0.00	0.00	0.00	0.00	8.25	1.65
		1-1	29H9C8_CD28	34.10	35.42	23.47	8.87	53.86	31.14
		1-2		6.56	4.89	0.15	0.00	26.41	7.60
		1-4		0.00	0.00	0.00	0.00	4.58	0.92
	72H	1-1	NT	0.00	0.00	0.00	0.00	11.68	2.34
		1-2		0.00	0.00	0.00	0.00	11.23	2.25
		1-4		0.00	0.00	0.00	0.00	6.14	1.23
		1-1	24A7G6_4-1BB	39.58	4.26	20.94	57.10	64.35	37.25
		1-2		26.25	0.00	12.95	32.06	30.79	20.41
		1-4		0.00	0.00	8.20	7.44	21.62	7.45
		1-1	29H9C8_4-1BB	48.13	0.00	34.26	52.14	47.17	36.34
		1-2		18.49	0.00	10.70	28.34	29.64	17.43
		1-4		0.00	0.00	4.72	0.29	26.14	6.23
		1-1	24A7G6_CD28	43.19	0.00	39.54	21.09	64.07	33.58
		1-2		0.00	0.00	11.50	0.00	27.84	7.87
		1-4		0.00	0.00	5.53	0.00	26.32	6.37
		1-1	29H9C8_CD28	45.81	34.78	11.77	43.83	60.37	39.31
		1-2		2.55	0.00	0.00	7.78	37.30	9.53
		1-4		0.00	0.00	0.00	0.00	21.89	4.38

**Group**			**CAR construct**	**Replicate**					**Mean**
				**N1**	**N2**	**N3**	**N4**	**N5**	
NALM6	24H	1-1	NT	0.00	7.80	13.97	17.22	0.00	7.80
		1-2		0.00	0.00	3.67	0.48	0.00	0.83
		1-4		0.00	0.00	1.86	0.00	0.00	0.37
		1-1	24A7G6_4-1BB	37.42	51.15	56.13	23.86	0.00	33.71
		1-2		15.62	22.46	43.45	21.41	9.37	22.46
		1-4		0.00	5.01	20.02	0.00	0.00	5.01
		1-1	29H9C8_4-1BB	57.35	26.05	61.35	21.71	0.00	33.29
		1-2		37.74	28.14	54.27	20.56	0.00	28.14
		1-4		27.50	17.25	31.60	9.91	0.00	17.25
		1-1	24A7G6_CD28	36.43	36.58	40.99	28.78	40.11	36.58
		1-2		6.42	12.91	32.50	4.40	8.30	12.91
		1-4		4.33	3.16	5.09	3.22	0.00	3.16
		1-1	29H9C8_CD28	41.03	39.73	52.50	27.10	38.27	39.73
		1-2		0.00	12.65	29.03	0.87	20.72	12.65
		1-4		0.00	5.81	23.23	0.00	0.00	5.81
	48H	1-1	NT	0.00	0.00	0.00	8.79	0.00	1.76
		1-2		0.00	0.00	0.00	10.83	0.00	2.17
		1-4		0.00	0.00	0.00	0.00	0.00	0.00
		1-1	24A7G6_4-1BB	74.68	61.12	62.74	43.79	63.28	61.12

		1-2		43.61	42.22	37.15	39.71	48.41	42.22
		1-4		27.86	25.97	26.64	29.88	19.50	25.97
		1-1	29H9C8_4-1BB	82.52	81.25	70.95	69.29	42.43	69.29
		1-2		73.04	48.70	58.75	50.67	22.18	50.67
		1-4		63.19	9.28	42.23	28.74	0.26	28.74
		1-1	24A7G6_CD28	53.73	56.32	36.92	55.38	79.25	56.32
		1-2		34.56	36.72	15.97	40.65	55.72	36.72
		1-4		24.56	19.78	0.00	24.41	30.15	19.78
		1-1	29H9C8_CD28	83.21	66.51	62.01	42.52	78.30	66.51
		1-2		55.56	39.82	30.30	26.78	46.63	39.82
		1-4		34.60	16.95	2.10	8.42	22.69	16.95
	72H	1-1	NT	0.00	0.00	12.35	9.99	4.07	5.28
		1-2		0.00	0.00	8.40	9.82	3.06	4.26
		1-4		0.00	0.00	7.32	7.40	3.68	3.68
		1-1	24A7G6_4-1BB	69.28	62.19	63.23	46.10	70.14	62.19
		1-2		35.41	31.12	29.77	26.59	38.70	32.32
		1-4		0.00	13.84	14.96	20.02	20.39	13.84
		1-1	29H9C8_4-1BB	81.63	62.27	70.46	40.05	56.93	62.27
		1-2		57.61	38.93	40.68	30.25	27.19	38.93
		1-4		13.07	18.73	31.54	15.85	14.46	18.73
		1-1	24A7G6_CD28	47.89	57.26	43.71	49.26	88.19	57.26
		1-2		10.60	30.60	18.61	36.74	56.47	30.60
		1-4		0.00	15.91	11.67	24.49	27.49	15.91
		1-1	29H9C8_CD28	73.69	62.68	43.84	47.84	85.37	62.68
		1-2		42.51	35.47	20.05	27.19	52.13	35.47
		1-4		0.00	11.84	18.20	11.04	18.11	11.84

**Group**			**CAR construct**	**Replicate**					**Mean**
				**N1**	**N2**	**N3**	**N4**	**N5**	
K562	24H	1-1	NT	0.00	0.00	0.00	0.00	0.00	0.00
		1-2		0.00	0.00	0.00	0.00	0.00	0.00
		1-4		0.00	0.00	0.00	0.00	0.00	0.00
		1-1	24A7G6_4-1BB	0.00	0.00	0.00	0.00	0.00	0.00
		1-2		0.00	0.00	0.00	0.00	0.00	0.00
		1-4		0.00	0.00	0.00	0.00	0.00	0.00
		1-1	29H9C8_4-1BB	0.00	0.00	0.00	0.00	0.00	0.00
		1-2		0.00	0.00	0.00	0.00	0.00	0.00
		1-4		0.00	0.00	0.00	0.00	0.00	0.00
		1-1	24A7G6_CD28	0.00	0.00	0.00	0.00	0.00	0.00
		1-2		0.00	0.00	0.00	0.00	0.00	0.00
		1-4		0.00	0.00	0.00	0.00	0.00	0.00
		1-1	29H9C8_CD28	0.00	0.00	0.00	0.00	0.00	0.00
		1-2		0.00	0.00	0.00	0.00	0.00	0.00
		1-4		0.00	0.00	0.00	0.00	0.00	0.00
	48H	1-1	NT	0.00	0.00	0.00	0.00	0.00	0.00
		1-2		0.00	0.00	0.00	0.00	0.00	0.00
		1-4		0.00	0.00	0.00	0.00	0.00	0.00
		1-1	24A7G6_4-1BB	12.18	0.00	7.31	4.00	9.00	6.50
		1-2		3.41	0.00	4.58	1.46	3.86	2.66
		1-4		0.00	0.00	1.47	0.09	0.89	0.49
		1-1		18.50	0.00	5.04	5.34	10.34	7.84

		1-2	29H9C8_4-1BB	5.76	0.00	3.70	1.96	4.35	3.15
		1-4		0.00	0.00	0.00	0.00	0.00	0.00
		1-1	24A7G6_CD28	4.92	11.27	2.94	3.88	8.86	6.37
		1-2		0.00	6.48	0.00	0.96	3.36	2.16
		1-4		0.00	0.00	0.00	0.00	0.00	0.00
		1-1	29H9C8_CD28	0.00	4.23	0.00	1.01	1.81	1.41
		1-2		0.00	0.00	0.00	0.00	0.00	0.00
		1-4		0.00	0.00	0.00	0.00	0.00	0.00
	72H	1-1	NT	0.00	0.00	0.00	0.00	0.00	0.00
		1-2		0.00	0.00	0.00	0.00	0.00	0.00
		1-4		0.00	0.00	0.00	0.00	0.00	0.00
		1-1	24A7G6_4-1BB	0.00	0.00	0.00	0.00	0.00	0.00
		1-2		0.00	0.00	0.00	0.00	0.00	0.00
		1-4		0.00	0.00	0.00	0.00	0.00	0.00
		1-1	29H9C8_4-1BB	12.78	7.59	0.00	6.79	4.79	6.39
		1-2		0.00	0.00	0.00	0.00	0.00	0.00
		1-4		0.00	0.00	0.00	0.00	0.00	0.00
		1-1	24A7G6_CD28	4.64	8.99	0.00	4.54	2.54	4.14
		1-2		0.00	0.00	0.00	0.00	0.00	0.00
		1-4		0.00	0.00	0.00	0.00	0.00	0.00
		1-1	29H9C8_CD28	0.00	4.30	0.00	1.43	0.43	1.23
		1-2		0.00	0.00	0.00	0.00	0.00	0.00
		1-4		0.00	0.00	0.00	0.00	0.00	0.00

Figure 5

A. CYTOKINE RELEASE (pg/ml)

Group	CAR construct	Replicate			Mean
		N1	N2	N3	
INF-y	NT	15.02	2.39	12.41	9.94
	24A7G6_4-1BB	48.01	28.66	21.63	32.77
	29H9C8_4-1BB	50.72	41.94	29.12	40.59
	24A7G6_CD28	31.28	36.07	44.05	37.13
	29H9C8_CD28	53.06	31.84	44.41	43.10
TNF	NT	3.54	1.37	1.40	2.10
	24A7G6_4-1BB	16.20	10.41	3.37	9.99
	29H9C8_4-1BB	19.50	10.51	3.78	11.26
	24A7G6_CD28	22.41	25.29	13.50	20.40
	29H9C8_CD28	36.90	19.90	33.57	30.12
IL-2	NT	2.12	1.79	2.10	2.00
	24A7G6_4-1BB	4.38	2.94	2.27	3.20
	29H9C8_4-1BB	3.74	3.08	2.30	3.04
	24A7G6_CD28	2.71	7.82	5.53	5.35
	29H9C8_CD28	4.00	4.58	9.77	6.12

IL-4	NT	2.25	1.24	1.13	1.54
	24A7G6_4-1BB	6.63	2.49	1.49	3.54
	29H9C8_4-1BB	7.68	3.09	1.59	4.12
	24A7G6_CD28	2.96	8.12	5.46	5.51
	29H9C8_CD28	5.91	6.31	3.83	5.35
IL-6	NT	18.63	1.90	6.23	8.92
	24A7G6_4-1BB	25.36	2.77	5.42	11.18
	29H9C8_4-1BB	27.08	1.89	6.03	11.67
	24A7G6_CD28	16.58	2.41	7.96	8.98
	29H9C8_CD28	22.38	2.73	6.07	10.39
IL-10	NT	1.51	1.46	2.03	1.67
	24A7G6_4-1BB	4.08	1.26	1.31	2.22
	29H9C8_4-1BB	1.94	0.97	1.49	1.47
	24A7G6_CD28	5.16	2.31	4.11	3.86
	29H9C8_CD28	3.33	2.39	3.71	3.14

Supplement 2

A. EXHAUSION (%)EXHAUSION

Group	CAR construct		Replicate					Mean
			N1	N2	N3	N4	N5	
TIGIT	NT	BEFORE	5.75	0.98	2.71	0.79	0.65	2.18
		AFTER	1.44	0.51	0.99	0.76	1.00	0.94
	24A7G6_4-1BB	BEFORE	6.94	0.53	3.31	0.09	0.54	2.28
		AFTER	7.10	5.67	7.66	0.43	2.69	4.71
	29H9C8_4-1BB	BEFORE	5.62	0.68	3.45	0.00	0.43	2.04
		AFTER	11.00	6.49	12.50	1.88	5.44	7.46
	24A7G6_CD28	BEFORE	7.22	0.23	3.67	0.12	0.62	2.37
		AFTER	13.60	9.48	13.60	0.55	3.39	8.12
	29H9C8_CD28	BEFORE	5.75	0.98	2.71	0.79	0.65	2.18
		AFTER	1.44	0.51	0.99	0.76	1.00	0.94
PD-1	NT	BEFORE	3.02	2.22	4.34	3.40	4.01	3.40
		AFTER	1.83	3.83	0.99	1.94	1.09	1.94
	24A7G6_4-1BB	BEFORE	2.73	2.01	2.78	1.36	2.54	2.28
		AFTER	1.76	3.33	7.66	3.29	0.80	3.37
	29H9C8_4-1BB	BEFORE	2.76	2.05	3.09	2.85	3.50	2.85
		AFTER	1.95	3.09	7.82	5.17	1.32	3.87
	24A7G6_CD28	BEFORE	5.05	1.98	4.46	2.88	5.32	3.94
		AFTER	10.10	9.24	12.50	8.58	2.47	8.58
	29H9C8_CD28	BEFORE	5.60	1.83	5.24	3.94	3.09	3.94
		AFTER	9.14	6.94	13.60	7.59	0.69	7.59
LAG3	NT	BEFORE	9.75	0.82	7.23	5.26	3.24	5.26

		AFTER	4.84	5.59	6.15	4.50	1.42	4.50
	24A7G6_4-1BB	BEFORE	25.10	8.68	5.64	11.04	4.76	11.04
		AFTER	7.89	10.60	5.08	6.97	4.30	6.97
	29H9C8_4-1BB	BEFORE	18.90	12.50	7.03	11.36	7.02	11.36
		AFTER	9.55	14.10	6.00	7.55	0.57	7.55
	24A7G6_CD28	BEFORE	14.10	12.30	4.60	8.32	3.21	8.51
		AFTER	8.32	22.30	13.10	24.80	12.20	16.14
	29H9C8_CD28	BEFORE	10.90	11.40	6.44	10.90	2.90	8.51
		AFTER	10.90	26.40	15.60	23.20	9.72	17.16
TIM3	NT	BEFORE	9.19	17.90	10.50	3.58	4.80	9.19
		AFTER	1.63	1.14	1.31	2.93	1.14	1.63
	24A7G6_4-1BB	BEFORE	16.20	19.20	24.50	7.58	13.50	16.20
		AFTER	12.36	9.14	9.62	20.00	10.70	12.36
	29H9C8_4-1BB	BEFORE	18.58	24.40	20.70	8.12	21.10	18.58
		AFTER	12.07	8.01	7.39	15.90	17.00	12.07
	24A7G6_CD28	BEFORE	13.54	17.40	12.70	5.75	18.30	13.54
		AFTER	20.62	10.50	9.78	38.80	23.40	20.62
	29H9C8_CD28	BEFORE	10.67	12.70	15.70	5.87	8.41	10.67
		AFTER	16.43	12.50	10.80	31.50	10.90	16.43

B. ACTIVATION (%)

**Group**	**CAR construct**		**Replicate**					**Mean**
			**N1**	**N2**	**N3**	N4	N5	
CD25	NT	BEFORE	97.30	95.60	95.10	97.90	75.40	92.26
		AFTER	37.20	87.50	81.80	86.90	42.40	67.16
	24A7G6_4-1BB	BEFORE	94.40	98.10	98.50	99.60	93.20	96.76
		AFTER	61.30	90.60	93.50	92.30	77.10	82.96
	29H9C8_4-1BB	BEFORE	96.10	96.10	97.40	99.70	91.60	96.18
		AFTER	67.30	87.80	91.10	91.90	77.20	83.06
	24A7G6_CD28	BEFORE	95.40	97.90	96.90	99.80	92.60	96.52
		AFTER	96.00	91.20	93.30	92.40	76.60	89.90
	29H9C8_CD28	BEFORE	95.40	97.20	97.70	99.60	89.70	95.92
		AFTER	76.30	90.60	89.50	88.30	71.20	83.18
CD69	NT	BEFORE	32.80	15.30	8.72	9.35	26.20	18.47
		AFTER	3.47	44.40	15.90	21.10	13.50	19.67
	24A7G6_4-1BB	BEFORE	13.60	15.60	16.90	17.70	24.30	17.62
		AFTER	4.23	10.30	24.50	28.10	28.60	19.15
	29H9C8_4-1BB	BEFORE	11.50	15.90	14.50	19.30	26.70	17.58
		AFTER	3.06	8.79	24.90	33.40	29.70	19.97
	24A7G6_CD28	BEFORE	9.87	17.20	11.80	16.20	27.50	16.51
		AFTER	9.12	9.48	27.50	41.90	30.50	23.70
	29H9C8_CD28	BEFORE	9.82	13.20	13.20	13.00	22.50	14.34
		AFTER	3.70	8.10	27.90	36.20	25.60	20.30

Figure. 6B Long term co-culture show number of Target cell (cells)																		
Day	Raji			NT			29H9C8_41BB			29H9C8_CD28			24A7G6_41BB			24A7G6_CD28		
0	1274	2222	1960	2010	2721	2121	1543	2848	2218	1496	2379	2247	1327	2360	1842	1290	2478	2085
1	4921	2230	1779	5155	2793	978	2520	2904	935	2207	2616	1646	2818	2531	981	3628	3648	927
4	31332	29156	22894	23413	30074	24263	2475	11123	8239	4804	12879	11184	5355	14265	8582	3168	7641	3949
7	29665	30567	22462	29476	29191	31315	7367	22481	11528	5294	19079	12607	10243	23869	11000	5170	7588	5262
12	28266.5	44644	31264	25908	46534	39692	5219.5	54313	38514	3425	52043	36950	11021.5	50857	43036	3179.5	19152	15456

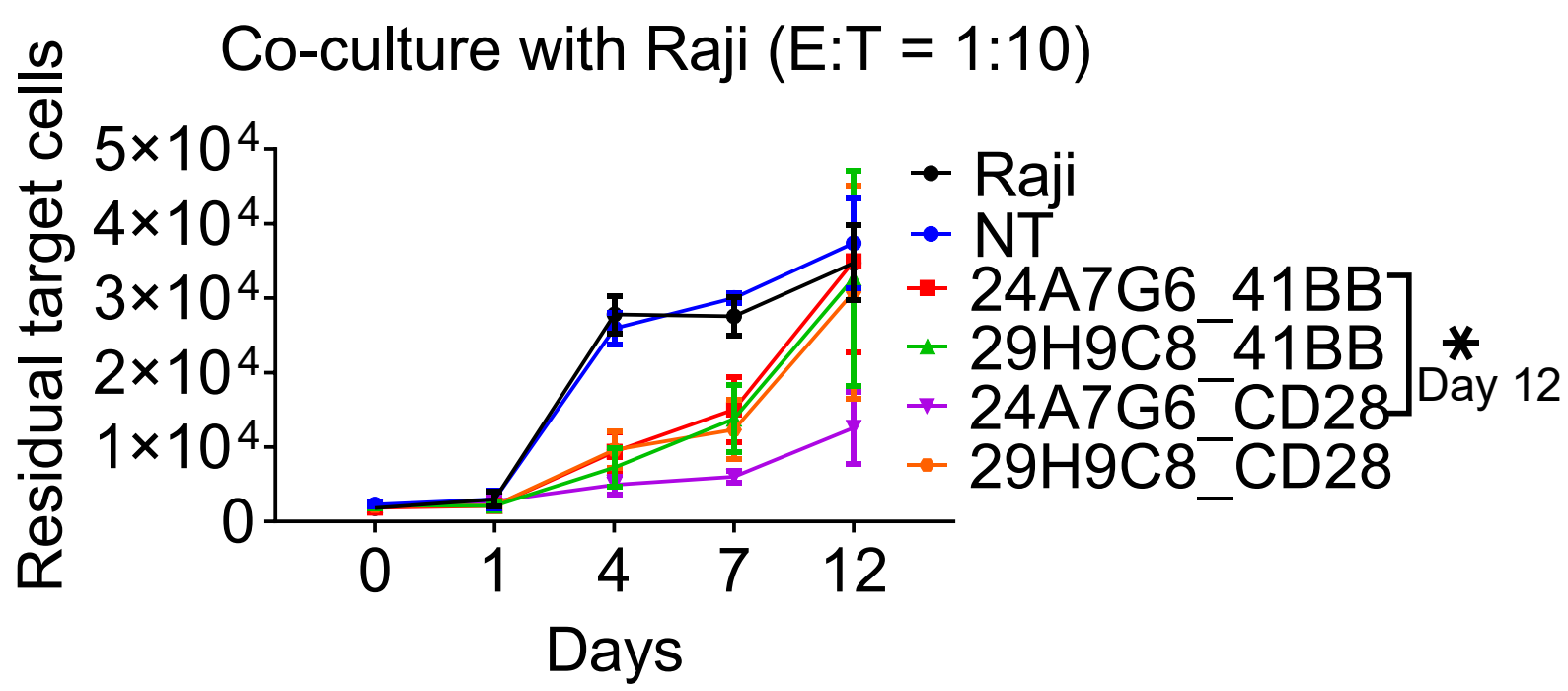

Figure. 6C Long term co-culture show number of Effector cell (cells)															
Day	NT			29H9C8_41BB			29H9C8_CD28			24A7G6_41BB			24A7G6_CD28		
0	197	265	584	942	313	382	845	225	263	548	251	329	817	474	515
1	275	255	241	1073	90	83	556	36	31	449	92	80	981	293	109
4	73	24	197	1462	100	761	1664	84	247	649	70	499	1522	343	1525
7	65	26	42	13615	420	1521	7644	189	1251	4502	123	562	12020	1152	3350
12	44	15	166	8420	472	1684	13544	875	2212	5782	149	1026	17404	1665	8908

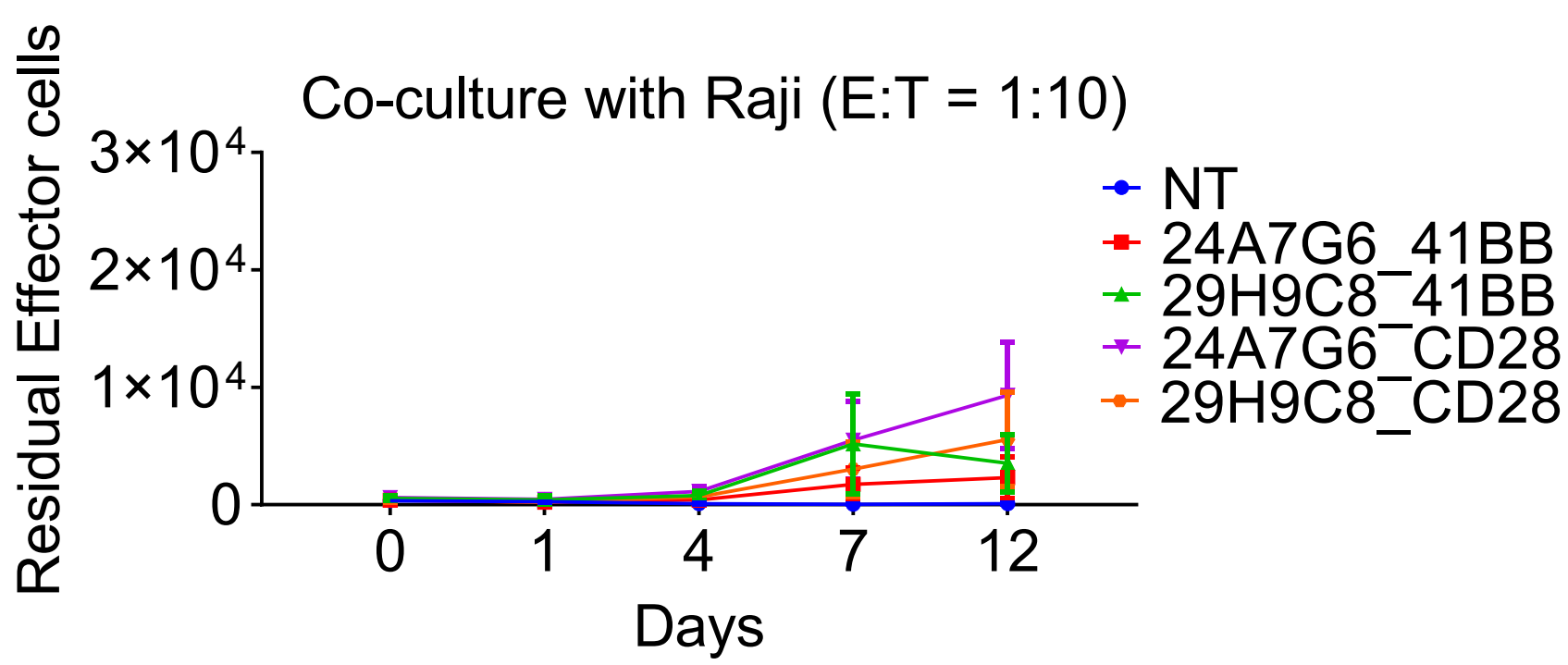

Figure. 6E Sequential co-culture show number of target cell (cells)

Round	Target			NT			29H9C8_41BB			29H9C8_CD28			24A7G6_41BB			24A7G6_CD28		
0	1000000	1000000	1000000	1000000	1000000	1000000	1000000	1000000	1000000	1000000	1000000	1000000	1000000	1000000	1000000	1000000	1000000	1000000
1	5314680	9561600	5179200	2861360	2703200	2528800	9044	55444	42828	11340	128520	58212	134976	38316	51088	6204	33600	32200
2	30300000	32900000	18507000	21432000	36472000	9979200	43428	825920	135720	35432	33228	124260	155944	25676	137888	35280	1543280	63656
3	56600000	48000000	27172800	41382400	71136000	34281400	74260	1551840	1290000	139776	6693	1671840	8138680	1921280	2041200	128960	17632	647072
4	59200000	64000000	65600000	90288000	1.19E+08	58915200	63180	5312160	6028800	20572	26028	4881800	13721400	3145520	5650200	34112	32064	1176600

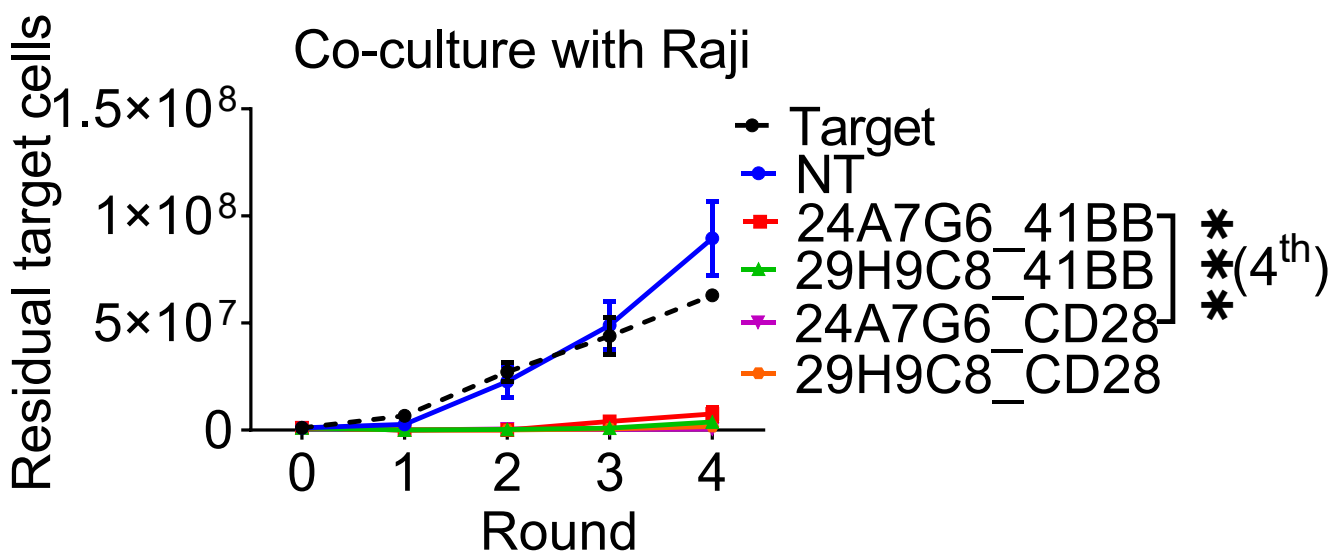

Figure. 6F Sequential co-culture show number of Effector cell (cells)

Round	NT			29H9C8_41BB			29H9C8_CD28			24A7G6_41BB			24A7G6_CD28		
0	4000000	4000000	4000000	11470000	6200000	7400000	7970000	5880000	5880000	6530000	5880000	7760000	7290000	8400000	6320000
1	609120	2659800	2488200	5245520	6432840	4917480	7476840	6317200	2861320	3969360	3552600	4736800	5527200	4656000	4462000
2	4959000	526400	427840	9055200	5738720	6584160	8091680	4628520	3998120	3591560	5061840	4573120	5703600	5825680	5641440
3	652960	338400	1111340	3039920	5444160	6338200	6945120	7070160	6833160	4397400	2930560	5065200	5840400	9159360	10161600
4	255360	168000	1203840	2379000	2618000	10617600	5409880	9447200	8885800	3306600	1474120	6964200	6284480	6492960	8564800

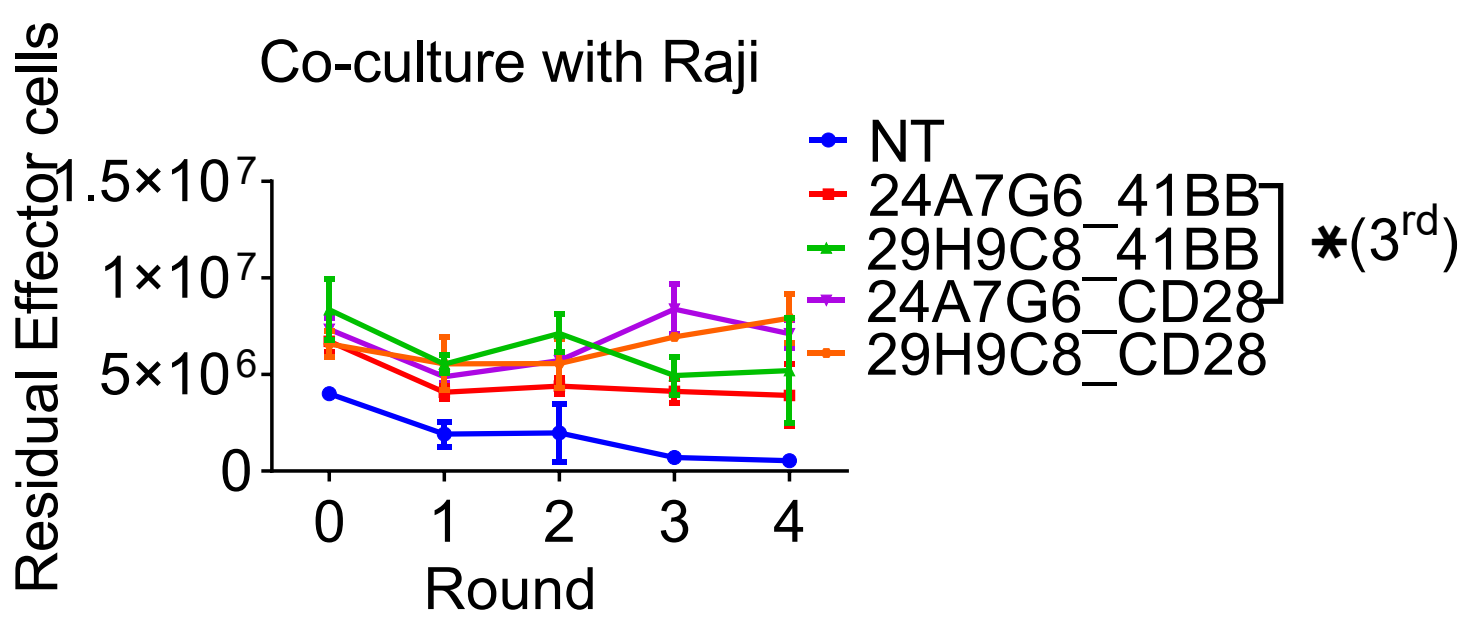

Supplement: Supplementary file 1 [file pone.0362221.s003.pdf]
